# Investigating genetic correlations and causal effects between caffeine consumption and sleep behaviours

**DOI:** 10.1111/jsr.12695

**Published:** 2018-04-22

**Authors:** Jorien L. Treur, Mark Gibson, Amy E. Taylor, Peter J. Rogers, Marcus R. Munafò

**Affiliations:** ^1^ School of Experimental Psychology University of Bristol Bristol UK; ^2^ MRC Integrative Epidemiology Unit (IEU) University of Bristol Bristol UK; ^3^ UK Centre for Tobacco and Alcohol Studies Bristol UK

**Keywords:** 1,3,7‐Trimethylxanthine, genetic overlap, duration of sleep, ‘morningness’, instrumental variable analysis, sleeplessness

## Abstract

Observationally, higher caffeine consumption is associated with poorer sleep and insomnia. We investigated whether these associations are a result of shared genetic risk factors and/or (possibly bidirectional) causal effects. Summary‐level data were available from genome‐wide association studies on caffeine intake (*n *=* *91 462), plasma caffeine and caffeine metabolic rate (*n *=* *9876), sleep duration and chronotype (being a “morning” versus an “evening” person) (*n *=* *128 266), and insomnia complaints (*n *=* *113 006). First, genetic correlations were calculated, reflecting the extent to which genetic variants influencing caffeine consumption and those influencing sleep overlap. Next, causal effects were estimated with bidirectional, two‐sample Mendelian randomization. This approach utilizes the genetic variants most robustly associated with an exposure variable as an “instrument” to test causal effects. Estimates from individual variants were combined using inverse‐variance weighted meta‐analysis, weighted median regression and MR‐Egger regression. We found no clear evidence for a genetic correlation between caffeine intake and sleep duration (*r*g = 0.000, *p *=* *.998), chronotype (*r*g = 0.086, *p *=* *.192) or insomnia complaints (*r*g = −0.034, *p *=* *.700). For plasma caffeine and caffeine metabolic rate, genetic correlations could not be calculated because of the small sample size. Mendelian randomization did not support causal effects of caffeine intake on sleep, or vice versa. There was weak evidence that higher plasma caffeine levels causally decrease the odds of being a morning person. Although caffeine may acutely affect sleep when taken shortly before bedtime, our findings suggest that a sustained pattern of high caffeine consumption is more likely to be associated with poorer sleep through shared environmental factors. Future research should identify such environments, which could aid the development of interventions to improve sleep.

## 1 INTRODUCTION

Caffeine is the most commonly used psychoactive substance, with coffee being the second most popular beverage worldwide (after water) (Butt & Sultan, 2011). There are also cultural differences in the popularity of caffeinated beverages, with tea being more popular than coffee in some countries, such as the UK (Treur et al., 2016). Acutely, caffeine is known to affect alertness and concentration through its antagonistic effects on adenosine receptors (Griffiths et al., 1990; Porkka‐Heiskanen & Kalinchuk, 2011), although because of tolerance the net benefit of frequent caffeine consumption appears to be negligible (Rogers, Heatherley, Mullings, & Smith, 2013). Consumption of caffeinated beverages has also been linked to poor sleep. A recent review of the literature showed that an average higher caffeine consumption is associated with prolonged sleep latency (the time it takes to fall asleep), reduced sleep time, reduced sleep efficiency (percentage of time asleep of the total time in bed) and poorer sleep quality (Clark & Landolt, 2017). Moreover, caffeine consumption correlates positively with insomnia complaints (Chaudhary, Grandner, Jackson, & Chakravorty, 2016; Skarupke et al., 2017) and negatively with chronotype (being a “morning” versus an “evening” person) (Fabbian et al., 2016; Suh et al., 2017). Given the higher mortality rates and poorer health outcomes associated with sleep problems (Itani, Jike, Watanabe, & Kaneita, 2017; Tang, Fiecas, Afolalu, & Wolke, 2017), it is important to understand how caffeine consumption relates to different sleep behaviours.

The co‐occurrence of high caffeine consumption and poor sleep may be the result of different (not mutually exclusive) mechanisms. First, factors that increase the amount of caffeine a person consumes may also increase their risk of having problems with sleeping. Such overlapping risk factors could be environmental in nature or genetic. From twin‐family studies, we know that caffeine consumption as well as sleep behaviours are heritable. Individual differences in caffeine consumption were explained by genetic factors for ~50% (Treur et al., 2017), whereas this was ~39% for sleep duration (Watson et al., 2016), ~42% for chronotype (Toomey, Panizzon, Kremen, Franz, & Lyons, 2015) and ~59% in women and ~38% in men for insomnia (Lind, Aggen, Kirkpatrick, Kendler, & Amstadter, 2015). More recently, large‐scale genome‐wide association (GWA) studies have identified specific genetic variants associated with each of these traits (Cornelis et al., 2015; Hammerschlag et al., 2017; Jones et al., 2016). Apart from overlapping (genetic) risk factors, the association between caffeine consumption and sleep may also be explained by causal effects. Given the well‐known stimulating effects of caffeine, it seems plausible that a sustained, high intake of caffeine can cause problems with sleeping. In extreme cases, it may even cause or exacerbate symptoms of insomnia. Controlled laboratory studies suggest that caffeine negatively impacts human sleep quality (Clark & Landolt, 2017). In these studies, however, caffeine was typically administered shortly before habitual bedtime (i.e. ≤60 min before), which may not reflect real‐life consumption patterns. In addition, most of these studies have been conducted in male participants only. More importantly, laboratory studies do not provide insight into the effects of prolonged high(er) intake of caffeine and causal effects in the other direction have not been tested: individuals who tend to sleep less and/or have insomnia may consume more caffeine to alleviate the effects of sleep deprivation during the day (Clark & Landolt, 2017; Penetar et al., 1993). Novel methods are needed to fully disentangle the complex relationship between caffeine consumption and sleep, focusing especially on possible longer‐term causal effects.

To determine whether observational associations between caffeine consumption and sleep variables are a result of overlapping genetic risk factors and/or causal effects (in either direction), we applied two methods. First, we calculated genetic correlations between caffeine consumption and sleep duration, insomnia complaints and chronotype based on summary level data of recent GWA studies (Cornelis et al., 2015, 2016; Hammerschlag et al., 2017; Jones et al., 2016). These genetic correlations reflect the extent to which genetic variants that are known to influence caffeine consumption also influence sleep behaviours (Bulik‐Sullivan et al., 2015). Evidence of genetic correlation indicates shared genetic aetiologies but may also (partly) reflect causal effects. If caffeine consumption causally affects sleep, one would expect that genetic variants that predict caffeine consumption, also predict sleep. To further investigate the possibility of such causal effects, and their direction, we applied two‐sample Mendelian randomization (MR) analysis. This instrumental variable approach utilizes a selection of genetic variants that are robustly associated with an exposure variable as an instrument to test causal effects on an outcome variable (Burgess, Scott, Timpson, Davey Smith, & Thompson, 2015; Davey Smith & Ebrahim, 2003). We examined potential biological pleiotropy (i.e.. effects of genotype on the outcome of interest not acting through the exposure) with two sensitivity analyses. By combining two novel research methods we aim to disentangle mechanisms underlying observational associations between caffeine consumption and sleep behaviours.

## 2 METHODS

### 2.1 Study population

To capture caffeine consumption, we used summary statistics from two different GWA studies. The first study was the Coffee and Caffeine Genetics Consortium GWA study (*n* = 91 462) (Cornelis et al., 2015), which investigated caffeine intake. The outcome of this study was cups of coffee per day, but genetic risk scores composed of the top genetic hits have been shown to be associated more generally with the intake of other types of caffeinated beverages (e.g. tea) as well (Taylor, Davey Smith, & Munafò, 2018). The second study was a GWA study that looked at plasma caffeine and its main metabolites as measured in the blood (*n *=* *9876) (Cornelis et al., 2016). The most informative outcomes of this GWA study were total plasma caffeine and the paraxanthine/plasma caffeine ratio, which reflects caffeine metabolic rate. Paraxanthine is the main metabolite of caffeine, with a higher ratio indicating a faster caffeine metabolism (Cornelis et al., 2016).

For sleep behaviours, GWA studies’ summary statistics were available for sleep duration, in hours of sleep and chronotype (a continuous score of being a “morning” versus an “evening” person) (both *n *=* *128 266) (Jones et al., 2016), and for insomnia complaints (usually having trouble falling asleep at night or waking up in the middle of the night [“cases”] versus never/rarely or sometimes having these problems [“controls”]) (*n *=* *113 006) (Hammerschlag et al., 2017). The GWA studies on sleep behaviours were performed using UK Biobank (Sudlow et al., 2015) and there was no sample overlap with the GWA studies on caffeine consumption.

### 2.2 LD score regression

To calculate genetic correlations between caffeine consumption and sleep behaviours, we employed LD score regression. Linkage disequilibrium (LD) is the degree to which genetic variants (single nucleotide polymorphisms [SNPs]) are transmitted together from parent to offspring. The main premise of LD score regression is that genetic variants that are in high LD with other genetic variants across the genome, are more likely to tag a causal genetic variant (one that exerts a true, causal effect on the phenotype in question) than genetic variants that are in low LD with other genetic variants. Based on this expected relationship between LD and the strength of association, for two phenotypes, a genetic correlation can be calculated. The genetic correlation reflects to what degree the genetic liability for one phenotype correlates with the genetic liability for a second phenotype. LD score regression methods have been described in more detail previously (Bulik‐Sullivan et al., 2015). We calculated genetic correlations using the summary data described above. Pre‐calculated and publicly available LD scores (the degree of LD an SNP has with all neighbouring SNPs) based on individuals of European ancestry were retrieved from https://github.com/bulik/ldsc.

### 2.3 Mendelian randomization

Mendelian randomization (MR) uses genetic variants that are robustly associated with an exposure variable as an instrument to test causal effects on an outcome variable (Davey Smith & Ebrahim, 2003). With conventional epidemiological methods, it is difficult to determine causality because an observational association can also be the result of confounding factors that predict both variables (e.g. socio‐economic position) or reverse causality (an outcome variable affecting the exposure variable). MR is in principle better protected against confounding than conventional epidemiological methods because genetic variants are randomly transmitted in the population. Additionally, reverse causality cannot affect MR results because an outcome variable cannot change a person's genotype. There are three important assumptions underlying MR: (i) the genetic instrument should be robustly associated with the exposure variable, (ii) the genetic instrument should be independent of confounders, and (iii) there should be no biological (or horizontal) pleiotropy, meaning that the genetic instrument should not affect the outcome variable through an independent pathway, other than through its effect on the exposure variable.

Here, we applied *two‐sample* MR, in which a genetic instrument is first identified in a GWA study of the exposure variable (*gene–exposure association*) and then the same instrument is identified in a second, separate GWA study of the outcome variable (*gene–outcome association*) (Burgess et al., 2015). When the genetic instrument was composed of a single genetic variant the Wald ratio method was applied (gene–outcome association/gene–exposure association) (Lawlor, Harbord, Sterne, Timpson, & Davey Smith, 2008). When the instrument comprised multiple genetic variants, Wald ratios were combined in an inverse‐variance weighted (IVW) meta‐analysis (summing ratio estimates of all variants in a weighted average formula) (Lawlor et al., 2008). To test the third MR assumption (no horizontal pleiotropy) we additionally used two sensitivity analyses. First, we used the weighted median approach, which is a method that can provide a consistent estimate of a causal effect even in a situation where up to 50% of the weight comes from invalid instruments (Bowden, Davey Smith, Haycock, & Burgess, 2016). Second, we used MR‐Egger regression, which applies Egger's test, normally used to assess small study bias in meta‐analyses, to genetic instruments with multiple genetic variants (Bowden, Davey Smith, & Burgess, 2015). Under MR‐Egger it is assumed that there is no correlation between the strength of an instrument (SNP–exposure association) and the effect that the instrument has on the outcome. This is referred to as the InSIDE assumption (instrument strength independent of direct effect) and it is a much weaker assumption than the assumption of no horizontal pleiotropy. MR‐Egger was only reported for genetic instruments that contained sufficient SNPs (≥10) (Bowden et al., 2015).

Genetic instruments were first identified for caffeine (caffeine intake, plasma caffeine and caffeine metabolic rate), after which causal effects on sleep behaviours (sleep duration, chronotype and insomnia complaints) were tested. Next, genetic instruments for the different sleep behaviours were identified and causal effects on caffeine were tested. For each phenotype, we constructed two genetic instruments: one consisting of SNPs that were associated with the exposure variable under the genome‐wide significant *p*‐value threshold of *p *<* *5 × 10^−8^ and one consisting of SNPs associated with the exposure variable under a more lenient *p*‐value threshold of *p *<* *1 × 10^−5^. All analyses were performed using the database and analytical platform *MR‐Base* (Hemani et al., 2016). For instruments of threshold *p *<* *5 × 10^−8^, all independent genome‐wide significant hits were selected manually from the published GWA study papers (based on the discovery samples) and then introduced to MR‐Base, whereas instruments of threshold *p *<* *1 × 10^−5^ were constructed in MR‐Base (including the pruning of genetic variants [*r* ^*2 *^< 0.001] and retrieving of proxies [*r* ^*2*^ **≥ 0.8]). Details of the SNPs included in all genetic instruments are provided in Table S1. Associations of SNPs with plasma caffeine and caffeine metabolic rate were only available as z‐scores, so we constructed beta coefficients and standard errors from the z‐scores, effect allele frequencies and sample size (see Tables 2 and 3 for the formula) (Taylor et al., 2016).

## 3 RESULTS

With LD score regression, we found no clear evidence for a genetic correlation between caffeine intake and sleep duration (*r*g = 0.000, standard error *[SE] *= 0.079, *p *=* *.998), caffeine intake and chronotype (*r*g = 0.086, *SE* = 0.066, *p *=* *.192), or caffeine intake and insomnia complaints (*r*g = −0.034, *SE *= 0.087, *p *=* *.700). Thus, across the whole genome, genetic variants that influence caffeine intake don't seem to be predictive of sleep behaviours. We were unable to calculate genetic correlations between plasma caffeine and caffeine metabolic rate and sleep behaviours, because of the modest sample size of the GWA studies these summary statistics were based on.

Two‐sample MR, using all three analytical approaches, did not provide clear evidence for causal effects of caffeine intake on sleep duration, chronotype or insomnia complaints, or vice versa. More details are provided in Table 1. Cochran's heterogeneity statistic (Q), which assesses heterogeneity between the different SNPs included in a genetic instrument, indicated heterogeneity for IVW analyses from caffeine intake to chronotype (see Table S2). The intercepts from MR‐Egger regression analyses, which estimate the degree of biological pleiotropy, did not provide strong evidence for pleiotropy overall, although there was some weak evidence for pleiotropy from chronotype to caffeine intake (see Table S3).

**Table 1:** Bidirectional, two‐sample Mendelian randomization analyses between caffeine intake and sleep behaviours

Exposure	Outcome	Threshold genetic instrument	*n* SNPs	Wald ratio/IVW				Weighted median				MR‐Egger			
				beta	OR	*SE*	*p*	beta	OR	*SE*	*p*	beta	OR	*SE*	*p*
Caffeine intake	Sleep duration	*p *< 5 × 10^−8^	4	−0.02		0.02	.337	−0.02		0.02	.492				
Caffeine intake	Sleep duration	*p *< 1 × 10^−5^	27	0.00		0.02	.796	0.01		0.02	.771	−0.01		0.03	.694
Caffeine intake	Chronotype	*p *< 5 × 10^−8^	4	0.03		0.03	.405	0.03		0.03	.228				
Caffeine intake	Chronotype	*p *< 1 × 10^−5^	27	−0.01		0.02	.743	0.00		0.02	.951	0.04		0.03	.207
Caffeine intake	Insomnia	*p *<* *5 × 10^−8^	4	−0.01	0.99	0.05	.856	0.00	1.00	0.05	.957				
Caffeine intake	Insomnia	*p *<* *1 × 10^−5^	27	−0.04	0.96	0.03	.168	−0.01	0.99	0.05	.890	−0.02	0.98	0.06	.712
Sleep duration	Caffeine intake	*p *<* *5 × 10^−8^	3	−0.12		0.17	.457	−0.14		0.19	.464				
Sleep duration	Caffeine intake	*p *<* *1 × 10^−5^	23	−0.15		0.10	.135	0.00		0.12	.987	0.41		0.37	.285
Chronotype	Caffeine intake	*p *<* *5 × 10^−8^	8	−0.01		0.12	.904	−0.11		0.15	.483				
Chronotype	Caffeine intake	*p *<* *1 × 10^−5^	55	0.09		0.06	.096	0.13		0.08	.092	−0.36		0.22	.113
Insomnia	Caffeine intake	*p *<* *5 × 10^−8^	1	0.07		0.13	.628								
Insomnia	Caffeine intake	*p *<* *1 × 10^−5^	16	−0.06		0.05	.194	−0.04		0.06	.515	−0.12		0.19	.554

In the case of a genetic instrument consisting of a single nucleotide polymorphism (SNP) the Wald ratio is reported, otherwise IVW (inverse‐variance weighted regression analysis) is reported. Weighted median regression analysis is only reported for genetic instruments consisting of ≥3 SNPs. MR‐Egger regression analysis is only reported for genetic instruments consisting of ≥10 SNPs. Definitions of the exposure and outcome variables in the genome‐wide association (GWA) studies were: caffeine intake (cups of coffee per day), sleep duration (hours of sleep), chronotype (a continuous score of being a ‘morning’ versus an ‘evening’ person) and insomnia (usually having trouble falling asleep at night or waking up in the middle of the night [‘cases’] versus never/rarely or sometimes having these problems [‘controls’]).

There was weak evidence that higher plasma caffeine levels decrease the odds of being a morning person (Wald ratio beta = −0.05, *p *=* *.045, and IVW beta = −0.03, *p *=* *.012, for genetic instruments with threshold *p *<* *5 × 10^−8^ and *p *<* *1 × 10^−5^, respectively; Table 2). The two sensitivity analyses indicated similar effect sizes in the same direction, albeit with weaker statistical evidence. There was also some weak evidence that insomnia complaints increase plasma caffeine, but only for the (one‐SNP) genetic instrument with threshold *p *<* *5 × 10^−8^ (Wald ratio beta = 0.47, *p *=* *.097). There was no clear evidence for other causal effects between plasma caffeine and sleep behaviours, nor was there evidence for heterogeneity between the different SNPs or biological pleiotropy (see Tables S4 and S5).

**Table 2:** Bidirectional, two‐sample Mendelian randomization analyses between plasma caffeine and sleep behaviours

Exposure	Outcome	Threshold genetic instrument	*n* SNPs	Wald ratio/IVW				Weighted median				MR‐Egger			
				beta	OR	*SE*	*p*	beta	OR	*SE*	*p*	beta	OR	*SE*	*p*
Plasma caffeine	Sleep duration	*p *<* *5 × 10^−8^	1	0.03		0.03	.285								
Plasma caffeine	Sleep duration	*p *<* *1 × 10^−5^	11	−0.01		0.01	.662	0.02		0.02	.355	−0.04		0.04	.367
Plasma caffeine	Chronotype	*p *<* *5 × 10^−8^	1	−0.05		0.03	.045								
Plasma caffeine	Chronotype	*p *<* *1 × 10^−5^	11	−0.03		0.01	.012	−0.03		0.02	.074	−0.05		0.05	.334
Plasma caffeine	Insomnia	*p *<* *5 × 10^−8^	1	0.02	1.02	0.06	.770								
Plasma caffeine	Insomnia	*p *<* *1 × 10^−5^	11	0.01	1.01	0.07	.340	0.02	1.02	0.04	.630	0.07	1.07	0.11	.556
Sleep duration	Plasma caffeine	*p *<* *5 × 10^−8^	2	0.25		0.54	.650								
Sleep duration	Plasma caffeine	*p *<* *1 × 10^−5^	16	0.34		0.27	.204	0.33		0.36	.355	0.71		1.08	.517
Chronotype	Plasma caffeine	*p *<* *5 × 10^−8^	4	0.05		0.49	.919	−0.08		0.57	.886				
Chronotype	Plasma caffeine	*p *<* *1 × 10^−5^	42	−0.22		0.17	.198	−0.38		0.24	.113	0.16		0.75	.834
Insomnia	Plasma caffeine	*p *<* *5 × 10^−8^	1	0.47		0.28	.097								
Insomnia	Plasma caffeine	*p *<* *1 × 10^−5^	14	0.07		0.13	.601	0.20		0.18	.248	0.32		0.52	.547

In the case of a genetic instrument consisting of a single nucleotide polymorphisms (SNP) the Wald ratio is reported, otherwise IVW (inverse‐variance weighted regression analysis) is reported. Weighted median regression analysis is only reported for genetic instruments consisting of ≥3 SNPs. MR‐Egger regression analysis is only reported for genetic instruments consisting of ≥10 SNPs. Definitions of the exposure and outcome variables in the genome‐wide association (GWA) studies were: plasma caffeine (caffeine levels as measured in blood plasma), sleep duration (hours of sleep), chronotype (a continuous score of being a ‘morning’ versus an ‘evening’ person) and insomnia (usually having trouble falling asleep at night or waking up in the middle of the night [‘cases’] versus never/rarely or sometimes having these problems [‘controls’]). For plasma caffeine, constructed beta values were calculated as Beta = z‐score/sqrt(N) * 1/SQRT(EAF(1‐EAF)). This calculation assumes that the standard errors are proportional to the inverse‐square root of the sample size multiplied by the variance of the genetic variant as a random variable (variance = EAF(1‐EAF)). This result should hold asymptotically.

There was weak evidence that a higher caffeine metabolic rate decreased sleep duration, but only for the genetic instrument with threshold *p *<* *1 × 10^−5^ (IVW beta = −0.02, *p *=* *0.045; Table 3). There was also some indication that a higher caffeine metabolic rate increases the odds of being a morning person, but only for the (one‐SNP) genetic instrument with threshold *p *<* *5 × 10^−8^ (Wald ratio beta = 0.04, *p *=* *.045). Finally, there was some weak evidence that a higher caffeine metabolic rate increases insomnia complaints, but only for the genetic instrument with threshold *p *<* *1 × 10^−5^ (IVW beta = 0.04, *p *=* *.057). There was no clear evidence for heterogeneity between SNPs, nor for biological pleiotropy (see Tables S6 and S7).

**Table 3:** Bidirectional, two‐sample Mendelian randomization analyses between caffeine metabolic rate and sleep behaviours

Exposure	Outcome	Threshold genetic instrument	*n* SNPs	Wald ratio/IVW				Weighted median				MR‐Egger			
				beta	OR	*SE*	*p*	beta	OR	*SE*	*p*	beta	OR	*SE*	*p*
Caffeine metabolic rate	Sleep duration	*p *<* *5 × 10^−8^	1	−0.02		0.02	0.285								
Caffeine metabolic rate	Sleep duration	*p *<* *1 × 10^−5^	8	−0.02		0.01	0.045	−0.02		0.01	0.150				
Caffeine metabolic rate	Chronotype	*p *<* *5 × 10^−8^	1	0.04		0.02	0.045								
Caffeine metabolic rate	Chronotype	*p *<* *1 × 10^−5^	8	0.01		0.01	0.547	0.03		0.01	0.074				
Caffeine metabolic rate	Insomnia	*p *<* *5 × 10^−8^	2	0.01	1.01	0.03	0.709								
Caffeine metabolic rate	Insomnia	*p *<* *1 × 10^−5^	9	0.04	1.04	0.02	0.057	0.02	1.02	0.03	0.492				
Sleep duration	Caffeine metabolic rate	*p *<* *5 × 10^−8^	2	−0.04		0.70	0.953								
Sleep duration	Caffeine metabolic rate	*p *<* *1 × 10^−5^	16	−0.17		0.35	0.624	−0.21		0.51	0.678	−0.57		1.45	0.699
Chronotype	Caffeine metabolic rate	*p *<* *5 × 10^−8^	4	0.26		0.63	0.686	0.16		0.73	0.822				
Chronotype	Caffeine metabolic rate	*p *<* *1 × 10^−5^	42	0.20		0.23	0.384	0.34		0.33	0.297	0.80		1.02	0.433
Insomnia	Caffeine metabolic rate	*p *<* *5 × 10^−8^	1	−0.57		0.36	0.118								
Insomnia	Caffeine metabolic rate	*p *<* *1 × 10^−5^	14	−0.09		0.18	0.609	−0.25		0.24	0.283	−0.33		0.73	0.658

In the case of a genetic instrument consisting of a single nucleotide polymorphism (SNP) the Wald ratio is reported, otherwise IVW (inverse‐variance weighted regression analysis) is reported. Weighted median regression analysis is only reported for genetic instruments consisting of ≥3 SNPs. MR‐Egger regression analysis is only reported for genetic instruments consisting of ≥10 SNPs. Definitions of the exposure and outcome variables in the genome‐wide association (GWA) studies were: caffeine metabolic rate (paraxanthine/plasma caffeine ratio, paraxanthine being the main metabolite of caffeine and the ratio reflecting an individual's metabolic rate of caffeine), sleep duration (hours of sleep), chronotype (a continuous score of being a ‘morning’ versus an ‘evening’ person) and insomnia (usually having trouble falling asleep at night or waking up in the middle of the night [‘cases’] versus never/rarely or sometimes having these problems [‘controls’]). For caffeine metabolic rate, constructed beta values were calculated as Beta = z‐score/sqrt(N) * 1/SQRT(EAF(1‐EAF)). This calculation assumes that the standard errors are proportional to the inverse‐square root of the sample size multiplied by the variance of the genetic variant as a random variable (variance = EAF(1‐EAF)). This result should hold asymptotically.

## 4 DISCUSSION

We did not find clear evidence in support of a genetic correlation between caffeine intake on the one hand and sleep duration, insomnia complaints or chronotype on the other hand. Apart from a few suggestive findings, which were further weakened as a result of the multiple testing burden, our results from Mendelian randomization analyses also did not support causal relationships from caffeine intake, plasma caffeine and caffeine metabolic rate to sleep behaviours, or the other way around. These results suggest that a longer‐term, average pattern of high caffeine consumption is associated with poorer sleep through shared environmental factors.

Our findings corroborate previous reports showing that none of the genetic variants associated with caffeine intake were associated with caffeine‐induced insomnia (Byrne et al., 2012; Cornelis et al., 2015). This might seem to contradict controlled laboratory studies that suggest that caffeine has a causal, negative impact on sleep (Clark & Landolt, 2017). However, in most of these studies, participants were administered caffeine immediately before their usual bedtime, and so *acute, short‐term* effects of caffeine were tested. In the current study, we measured genetic liability for caffeine intake, a measure that reflects a more *sustained life‐time* average intake of caffeine, and not only intake just before going to sleep. It may be the case that caffeine impacts sleep when it is consumed in the evening, whereas there is little or no effect when it is consumed during the day. It is likely that most caffeine is consumed earlier during the day, given that a common reason for consuming caffeinated beverages is their stimulant effects (Ludden, O'Brien, & Pasch, 2017; Reich, Dietrich, Reid Finlayson, Fischer, & Martin, 2008). One small study (*n *=* *12) looked at the effects of a high dose of caffeine (400 mg, similar to the amount of caffeine in at least four cups of coffee) on sleep when administered 0, 3 or 6 hr before bedtime and did find disruptive effects on sleep at all time‐points (Drake, Roehrs, Shambroom, & Roth, 2013). Another possibility for the lack of evidence for causal effects in the present study is that, over time, tolerance of the effects of caffeine develops (Rogers et al., 2013), which would mean that frequent consumption of caffeine doesn't disrupt sleep. In fact, caffeine withdrawal has previously been found to increase sleepiness, at least for daytime sleepiness (Rogers et al., 2013).

The most compelling of our suggestive causal findings was a negative effect of plasma caffeine levels on chronotype, decreasing the odds of being a morning person. This might indicate that people with higher circulating levels of caffeine stay up later and consequently find it harder to get up early in the morning. This is consistent with previous literature showing that being more of an evening person is associated with consuming more coffee and other caffeinated beverages (Fabbian et al., 2016; Suh et al., 2017). Caffeine levels measured in blood plasma should provide a more accurate measure of a person's exposure to the stimulating effects of caffeine because this considers biological differences in caffeine metabolism. However, our evidence was weak, and further research into this relationship is warranted.

In contrast to previous (laboratory) studies, we were also able to test causal effects in the direction from sleep behaviours to caffeine. We did not find any clear evidence for causal effects. This is in contrast to research showing that a common reason for changing coffee consumption is experiencing sleep problems (Soroko, Chang, & Barrett‐Connor, 1996). It may be that such causal effects did not emerge in our analyses because these are only short‐term adjustments in caffeine use that do not hold in the longer term, whereas our genetic approach reflects a longer‐term measure of caffeine consumption.

The lack of evidence for genetic correlation between caffeine consumption and sleep behaviours, and for causal effects, suggests that observational associations may be the result of shared environmental factors. The literature on this topic is scarce, but an example of an environmental factor that could be responsible for both increasing caffeine consumption and inducing or exacerbating sleeping problems is work or school‐related demands and stress (Dorrian et al., 2011; Zunhammer, Eichhammer, & Busch, 2014). Daily stress may cause people to have trouble sleeping and may consequently cause them to attempt to self‐medicate by consuming more caffeine. More research is needed to identify the environmental factors that increase both caffeine consumption and sleeping problems, in order to guide the development of more evidence‐based interventions to improve sleep.

A major strength of our approach, using summary‐level data of very large sample sizes, is that it provides considerable power to detect small effects, which are likely for complex traits such as caffeine consumption and sleep behaviours. There are also limitations to consider. For the Mendelian randomization analyses we assumed the caffeine consumption SNPs (Cornelis et al., 2015) to be associated with caffeine intake in the GWA study of the sleeping variables, but we were not able to test this. The genetic instrument may be weaker if the GWA study of the outcome variable contains a group of people that do not consume coffee. However, we have previously shown that the genetic risk score of caffeine consumption also predicts coffee consumption in the combined sample of coffee and non‐coffee drinkers in UK Biobank (Taylor et al., 2018). Another limitation is that, for plasma caffeine and caffeine metabolic rate, we were not able to calculate genetic correlations with sleep behaviours, because of the relatively low sample size in the GWA studies. In addition, the beta coefficients resulting from the plasma caffeine/caffeine metabolic rate MR analyses don't have interpretable units, given that SNP associations were constructed from z‐scores. We can, however, interpret the direction of effect and strength of the evidence (*p*‐values) for these analyses. For sleep behaviours, we relied on self‐reported measures, although self‐perceived sleep duration can be influenced by many other factors and may not fully reflect actual sleep. We also did not consider other sleep problems, such as sleep‐disordered breathing (apnea). Finally, it is important to note that the relationship between caffeine consumption and sleep is complex and we may not have been able to address all the complexities (e.g. timing of caffeine consumption and biological factors related to caffeine metabolism).

In summary, we did not find clear evidence of causal effects of caffeine consumption on sleep behaviours, or vice versa. Our findings provide new and relevant insights into the link between caffeine consumption and sleep, by showing that a sustained high consumption of caffeine doesn't seem to increase the risk of developing sleep problems. There also doesn't seem to be a shared genetic architecture between caffeine intake and sleep. These results highlight the complexity of interpreting Mendelian randomization results for health behaviours such as caffeine consumption and sleep. Although there are well‐known acute effects of caffeine on alertness this did not translate into strong evidence for causal effects of a more sustained intake of caffeine on sleep. Researchers applying Mendelian randomization should be aware that genetic variants used as an instrument, or proxy, for an (exposure) variable, reflect a lifetime exposure to higher or lower levels of that variable.

## AUTHOR CONTRIBUTIONS

MG and JLT carried out the analyses and drafted the manuscript. AET, PJR and MRM assisted with writing and interpretation of the findings. All authors reviewed the content of the manuscript and approved the final version.

## CONFLICT OF INTERESTS

JLT, MG, AET, PJR and MRM have no financial or non‐financial conflicts of interest to declare.
