# Supplementary material for: Investigating genetic correlations and causal effects between caffeine consumption and sleep behaviours
Source: J Sleep Res. 2018 Apr 22;27(5):e12695. doi: 10.1111/jsr.12695 (PMC6175249; doi:10.1111/jsr.12695)
Supplement: Supplementary file 1 [file JSR-27-na-s001.docx]

**Supplementary material**

**Table S1.** Single nucleotide polymorphisms (SNPs) included in the genetic instruments used for bi-directional, two sample Mendelian randomization analyses between caffeine consumption (caffeine intake, plasma caffeine and caffeine metabolic rate) and sleep behaviours (sleep duration, chronotype and insomnia complaints).

|  |  |  |  |  |  |  | Gene-exposure estimate | | | Gene-outcome estimate | | |
| --- | --- | --- | --- | --- | --- | --- | --- | --- | --- | --- | --- | --- |
| Exposure | Outcome | Threshold genetic instrument | SNP | Original SNP if proxy used (LD R^2^ with original SNP) | Effect allele | Effect allele frequency | beta | SE | p-value | beta | SE | p-value |
| Caffeine intake | Sleep duration | p<5×10^-8^ | rs17685 |  | A | 0.290 | 0.069 | 0.009 | 9.06E-14 | 0.002 | 0.004 | 0.660 |
|  |  |  | rs2470893 |  | T | 0.305 | 0.122 | 0.009 | 6.89E-44 | 0.000 | 0.004 | 0.940 |
|  |  |  | rs4410790 |  | T | 0.371 | -0.138 | 0.009 | 1.48E-57 | 0.005 | 0.004 | 0.260 |
|  |  |  | rs7800944 |  | T | 0.716 | -0.052 | 0.009 | 7.82E-09 | 0.007 | 0.004 | 0.130 |
| Caffeine intake | Sleep duration | p<1×10^-5^ | rs10007278 |  | T | 0.152 | 0.053 | 0.012 | 4.50E-06 | 0.000 | 0.005 | 0.960 |
|  |  |  | rs11863088 |  | A | 0.037 | 0.217 | 0.033 | 3.78E-11 | -0.020 | 0.010 | 0.061 |
|  |  |  | rs11977801 |  | T | 0.909 | -0.071 | 0.016 | 4.72E-06 | -0.007 | 0.007 | 0.350 |
|  |  |  | rs1260326 |  | T | 0.415 | -0.041 | 0.008 | 1.06E-07 | 0.005 | 0.004 | 0.180 |
|  |  |  | rs1481012 |  | A | 0.891 | 0.063 | 0.013 | 1.13E-06 | 0.004 | 0.006 | 0.550 |
|  |  |  | rs1571536 |  | T | 0.486 | -0.036 | 0.008 | 2.32E-06 | -0.006 | 0.004 | 0.130 |
|  |  |  | rs17685 |  | A | 0.290 | 0.069 | 0.009 | 9.06E-14 | 0.002 | 0.004 | 0.660 |
|  |  |  | rs2049045 |  | C | 0.189 | -0.051 | 0.010 | 2.08E-07 | 0.000 | 0.005 | 0.980 |
|  |  |  | rs2235852 |  | T | 0.317 | -0.042 | 0.008 | 5.94E-07 | -0.005 | 0.004 | 0.290 |
|  |  |  | rs2264517 |  | A | 0.319 | 0.040 | 0.009 | 2.85E-06 | -0.002 | 0.004 | 0.630 |
|  |  |  | rs2472297 |  | T | 0.241 | 0.146 | 0.010 | 6.45E-47 | 0.001 | 0.004 | 0.770 |
|  |  |  | rs2597398 |  | T | 0.460 | -0.036 | 0.008 | 2.56E-06 | -0.003 | 0.004 | 0.440 |
|  |  |  | rs2668822 |  | T | 0.600 | 0.037 | 0.008 | 1.96E-06 | -0.006 | 0.004 | 0.130 |
|  |  |  | rs347306 |  | T | 0.445 | -0.039 | 0.008 | 2.14E-06 | 0.004 | 0.004 | 0.300 |
|  |  |  | rs4245116 |  | T | 0.467 | 0.038 | 0.008 | 1.54E-06 | 0.000 | 0.004 | 0.950 |
|  |  |  | rs4410790 |  | T | 0.371 | -0.138 | 0.009 | 1.48E-57 | 0.005 | 0.004 | 0.260 |
|  |  |  | rs4907945 |  | T | 0.035 | 0.140 | 0.031 | 5.52E-06 | -0.012 | 0.011 | 0.260 |
|  |  |  | rs494620 |  | A | 0.425 | -0.035 | 0.008 | 8.13E-06 | -0.001 | 0.004 | 0.870 |
|  |  |  | rs6745468 |  | C | 0.827 | 0.051 | 0.011 | 4.75E-06 | -0.011 | 0.006 | 0.051 |
|  |  |  | rs7149225 |  | T | 0.158 | 0.077 | 0.017 | 5.51E-06 | 0.007 | 0.005 | 0.160 |
|  |  |  | rs738820 |  | T | 0.840 | 0.056 | 0.011 | 1.02E-06 | 0.012 | 0.006 | 0.032 |
|  |  |  | rs7605062 |  | A | 0.963 | -0.224 | 0.036 | 5.44E-10 | -0.003 | 0.013 | 0.840 |
|  |  |  | rs767778 |  | A | 0.635 | -0.038 | 0.008 | 6.29E-06 | -0.003 | 0.004 | 0.430 |
|  |  |  | rs7800944 |  | T | 0.716 | -0.052 | 0.009 | 7.82E-09 | 0.007 | 0.004 | 0.130 |
|  |  |  | rs8142427 |  | C | 0.968 | -0.243 | 0.047 | 1.82E-07 | -0.012 | 0.011 | 0.290 |
|  |  |  | rs9320679 |  | A | 0.107 | 0.063 | 0.013 | 1.60E-06 | 0.007 | 0.006 | 0.300 |
|  |  |  | rs9902453 |  | A | 0.537 | -0.036 | 0.008 | 2.26E-06 | 0.001 | 0.004 | 0.840 |
| Caffeine intake | Chronotype | p<5×10^-8^ | rs17685 |  | A | 0.290 | 0.069 | 0.009 | 9.06E-14 | 0.001 | 0.004 | 0.910 |
|  |  |  | rs2470893 |  | T | 0.305 | 0.122 | 0.009 | 6.89E-44 | 0.001 | 0.004 | 0.810 |
|  |  |  | rs4410790 |  | T | 0.371 | -0.138 | 0.009 | 1.48E-57 | -0.009 | 0.004 | 0.026 |
|  |  |  | rs7800944 |  | T | 0.716 | -0.052 | 0.009 | 7.82E-09 | 0.008 | 0.004 | 0.061 |
| Caffeine intake | Chronotype | p<1×10^-5^ | rs10007278 |  | T | 0.152 | 0.053 | 0.012 | 4.50E-06 | 0.003 | 0.005 | 0.550 |
|  |  |  | rs11863088 |  | A | 0.037 | 0.217 | 0.033 | 3.78E-11 | -0.005 | 0.010 | 0.610 |
|  |  |  | rs11977801 |  | T | 0.909 | -0.071 | 0.016 | 4.72E-06 | 0.002 | 0.007 | 0.770 |
|  |  |  | rs1260326 |  | T | 0.415 | -0.041 | 0.008 | 1.06E-07 | -0.005 | 0.004 | 0.260 |
|  |  |  | rs1481012 |  | A | 0.891 | 0.063 | 0.013 | 1.13E-06 | 0.004 | 0.006 | 0.490 |
|  |  |  | rs1571536 |  | T | 0.486 | -0.036 | 0.008 | 2.32E-06 | -0.009 | 0.004 | 0.031 |
|  |  |  | rs17685 |  | A | 0.290 | 0.069 | 0.009 | 9.06E-14 | 0.001 | 0.004 | 0.910 |
|  |  |  | rs2049045 |  | C | 0.189 | -0.051 | 0.010 | 2.08E-07 | 0.014 | 0.005 | 0.005 |
|  |  |  | rs2235852 |  | T | 0.317 | -0.042 | 0.008 | 5.94E-07 | 0.009 | 0.004 | 0.033 |
|  |  |  | rs2264517 |  | A | 0.319 | 0.040 | 0.009 | 2.85E-06 | -0.007 | 0.004 | 0.110 |
|  |  |  | rs2472297 |  | T | 0.241 | 0.146 | 0.010 | 6.45E-47 | -0.002 | 0.004 | 0.680 |
|  |  |  | rs2597398 |  | T | 0.460 | -0.036 | 0.008 | 2.56E-06 | -0.004 | 0.004 | 0.330 |
|  |  |  | rs2668822 |  | T | 0.600 | 0.037 | 0.008 | 1.96E-06 | 0.003 | 0.004 | 0.480 |
|  |  |  | rs347306 |  | T | 0.445 | -0.039 | 0.008 | 2.14E-06 | 0.010 | 0.004 | 0.015 |
|  |  |  | rs4245116 |  | T | 0.467 | 0.038 | 0.008 | 1.54E-06 | -0.001 | 0.004 | 0.860 |
|  |  |  | rs4410790 |  | T | 0.371 | -0.138 | 0.009 | 1.48E-57 | -0.009 | 0.004 | 0.026 |
|  |  |  | rs4907945 |  | T | 0.035 | 0.140 | 0.031 | 5.52E-06 | 0.004 | 0.010 | 0.690 |
|  |  |  | rs494620 |  | A | 0.425 | -0.035 | 0.008 | 8.13E-06 | 0.001 | 0.004 | 0.820 |
|  |  |  | rs6745468 |  | C | 0.827 | 0.051 | 0.011 | 4.75E-06 | -0.012 | 0.006 | 0.031 |
|  |  |  | rs7149225 |  | T | 0.158 | 0.077 | 0.017 | 5.51E-06 | 0.001 | 0.005 | 0.900 |
|  |  |  | rs738820 |  | T | 0.840 | 0.056 | 0.011 | 1.02E-06 | -0.003 | 0.005 | 0.550 |
|  |  |  | rs7605062 |  | A | 0.963 | -0.224 | 0.036 | 5.44E-10 | 0.000 | 0.013 | 0.990 |
|  |  |  | rs767778 |  | A | 0.635 | -0.038 | 0.008 | 6.29E-06 | 0.008 | 0.004 | 0.055 |
|  |  |  | rs7800944 |  | T | 0.716 | -0.052 | 0.009 | 7.82E-09 | 0.008 | 0.004 | 0.061 |
|  |  |  | rs8142427 |  | C | 0.968 | -0.243 | 0.047 | 1.82E-07 | -0.011 | 0.011 | 0.320 |
|  |  |  | rs9320679 |  | A | 0.107 | 0.063 | 0.013 | 1.60E-06 | -0.006 | 0.006 | 0.350 |
|  |  |  | rs9902453 |  | A | 0.537 | -0.036 | 0.008 | 2.26E-06 | 0.002 | 0.004 | 0.650 |
| Caffeine intake | Insomnia | p<5×10^-8^ | rs17685 |  | A | 0.290 | 0.069 | 0.009 | 9.06E-14 | 0.002 | 0.010 | 0.869 |
|  |  |  | rs2470893 |  | T | 0.305 | 0.122 | 0.009 | 6.89E-44 | 0.002 | 0.010 | 0.873 |
|  |  |  | rs4410790 |  | T | 0.371 | -0.138 | 0.009 | 1.48E-57 | 0.001 | 0.010 | 0.923 |
|  |  |  | rs7800944 |  | T | 0.716 | -0.052 | 0.009 | 7.82E-09 | 0.011 | 0.010 | 0.278 |
| Caffeine intake | Insomnia | p<1×10^-5^ | rs10007278 |  | T | 0.152 | 0.053 | 0.012 | 4.50E-06 | -0.004 | 0.013 | 0.731 |
|  |  |  | rs11863088 |  | A | 0.037 | 0.217 | 0.033 | 3.78E-11 | -0.006 | 0.025 | 0.806 |
|  |  |  | rs11977801 |  | T | 0.909 | -0.071 | 0.016 | 4.72E-06 | -0.021 | 0.017 | 0.207 |
|  |  |  | rs1260326 |  | T | 0.415 | -0.041 | 0.008 | 1.06E-07 | -0.001 | 0.010 | 0.906 |
|  |  |  | rs1481012 |  | A | 0.891 | 0.063 | 0.013 | 1.13E-06 | 0.008 | 0.015 | 0.583 |
|  |  |  | rs1571536 |  | T | 0.486 | -0.036 | 0.008 | 2.32E-06 | -0.005 | 0.010 | 0.579 |
|  |  |  | rs17685 |  | A | 0.290 | 0.069 | 0.009 | 9.06E-14 | 0.002 | 0.010 | 0.869 |
|  |  |  | rs2049045 |  | C | 0.189 | -0.051 | 0.010 | 2.08E-07 | 0.019 | 0.012 | 0.121 |
|  |  |  | rs2235852 |  | T | 0.317 | -0.042 | 0.008 | 5.94E-07 | 0.008 | 0.010 | 0.444 |
|  |  |  | rs2264517 |  | A | 0.319 | 0.040 | 0.009 | 2.85E-06 | -0.001 | 0.010 | 0.893 |
|  |  |  | rs2472297 |  | T | 0.241 | 0.146 | 0.010 | 6.45E-47 | 0.000 | 0.011 | 0.987 |
|  |  |  | rs2597398 |  | T | 0.460 | -0.036 | 0.008 | 2.56E-06 | 0.009 | 0.009 | 0.346 |
|  |  |  | rs2668822 |  | T | 0.600 | 0.037 | 0.008 | 1.96E-06 | 0.006 | 0.010 | 0.507 |
|  |  |  | rs347306 |  | T | 0.445 | -0.039 | 0.008 | 2.14E-06 | 0.001 | 0.009 | 0.957 |
|  |  |  | rs4245116 |  | T | 0.467 | 0.038 | 0.008 | 1.54E-06 | -0.002 | 0.009 | 0.871 |
|  |  |  | rs4410790 |  | T | 0.371 | -0.138 | 0.009 | 1.48E-57 | 0.001 | 0.010 | 0.923 |
|  |  |  | rs4907945 |  | T | 0.035 | 0.140 | 0.031 | 5.52E-06 | -0.028 | 0.025 | 0.271 |
|  |  |  | rs494620 |  | A | 0.425 | -0.035 | 0.008 | 8.13E-06 | 0.008 | 0.010 | 0.402 |
|  |  |  | rs6745468 |  | C | 0.827 | 0.051 | 0.011 | 4.75E-06 | -0.004 | 0.013 | 0.743 |
|  |  |  | rs7149225 |  | T | 0.158 | 0.077 | 0.017 | 5.51E-06 | -0.032 | 0.012 | 0.008 |
|  |  |  | rs738820 |  | T | 0.840 | 0.056 | 0.011 | 1.02E-06 | -0.017 | 0.013 | 0.203 |
|  |  |  | rs7605062 |  | A | 0.963 | -0.224 | 0.036 | 5.44E-10 | 0.043 | 0.031 | 0.171 |
|  |  |  | rs767778 |  | A | 0.635 | -0.038 | 0.008 | 6.29E-06 | 0.003 | 0.010 | 0.751 |
|  |  |  | rs7800944 |  | T | 0.716 | -0.052 | 0.009 | 7.82E-09 | 0.011 | 0.010 | 0.278 |
|  |  |  | rs8142427 |  | C | 0.968 | -0.243 | 0.047 | 1.82E-07 | -0.019 | 0.026 | 0.481 |
|  |  |  | rs9320679 |  | A | 0.107 | 0.063 | 0.013 | 1.60E-06 | 0.006 | 0.015 | 0.710 |
|  |  |  | rs9902453 |  | A | 0.537 | -0.036 | 0.008 | 2.26E-06 | -0.008 | 0.009 | 0.405 |
| Sleep duration | Caffeine intake | p<5×10^-8^ | rs1380703 |  | A | 0.618 | 0.025 | 0.004 | 7.60E-09 | -0.010 | 0.009 | 0.252 |
|  |  |  | rs17190618 |  | A | 0.840 | -0.033 | 0.005 | 1.20E-09 | 0.009 | 0.011 | 0.416 |
|  |  |  | rs1807282 | rs62158211 (0.99) | A | 0.788 | -0.039 | 0.005 | 8.70E-16 | -0.003 | 0.009 | 0.750 |
| Sleep duration | Caffeine intake | p<1×10^-5^ | rs10510128 |  | G | 0.795 | -0.023 | 0.005 | 4.60E-06 | -0.003 | 0.009 | 0.788 |
|  |  |  | rs10840160 |  | T | 0.536 | 0.019 | 0.004 | 2.70E-06 | -0.015 | 0.008 | 0.049 |
|  |  |  | rs11152363 |  | G | 0.814 | 0.023 | 0.005 | 8.30E-06 | -0.015 | 0.010 | 0.146 |
|  |  |  | rs11964802 | rs67059016 (1.0) | A | 0.712 | 0.019 | 0.004 | 8.50E-06 | 0.005 | 0.011 | 0.627 |
|  |  |  | rs1204056 | rs7797160 (0.88) | G | 0.338 | 0.015 | 0.004 | 3.00E-04 | 0.004 | 0.008 | 0.658 |
|  |  |  | rs12984777 |  | C | 0.972 | -0.058 | 0.012 | 2.10E-06 | -0.038 | 0.032 | 0.229 |
|  |  |  | rs1380703 |  | A | 0.618 | 0.025 | 0.004 | 7.60E-09 | -0.010 | 0.009 | 0.252 |
|  |  |  | rs16865859 | rs35684737 (0.99) | T | 0.870 | -0.027 | 0.006 | 3.20E-06 | 0.009 | 0.011 | 0.433 |
|  |  |  | rs16949934 |  | G | 0.920 | -0.034 | 0.007 | 3.30E-06 | -0.026 | 0.013 | 0.050 |
|  |  |  | rs17121264 |  | A | 0.926 | 0.036 | 0.008 | 5.00E-06 | 0.000 | 0.016 | 0.996 |
|  |  |  | rs17169082 | rs35188382 (0.96) | G | 0.920 | -0.032 | 0.007 | 1.00E-05 | -0.006 | 0.015 | 0.676 |
|  |  |  | rs17190618 |  | A | 0.840 | -0.033 | 0.005 | 1.20E-09 | 0.009 | 0.011 | 0.416 |
|  |  |  | rs1807282 | rs62158211 (0.99) | A | 0.788 | -0.039 | 0.005 | 8.70E-16 | -0.003 | 0.009 | 0.750 |
|  |  |  | rs242717 | rs62099775 (1.0) | T | 0.682 | -0.019 | 0.004 | 8.00E-06 | 0.002 | 0.008 | 0.786 |
|  |  |  | rs2846581 |  | C | 0.275 | -0.024 | 0.005 | 1.20E-07 | 0.013 | 0.008 | 0.120 |
|  |  |  | rs3095508 |  | C | 0.592 | 0.019 | 0.004 | 3.60E-06 | 0.003 | 0.008 | 0.670 |
|  |  |  | rs342745 |  | A | 0.201 | -0.023 | 0.005 | 5.40E-06 | -0.001 | 0.010 | 0.950 |
|  |  |  | rs6425885 |  | G | 0.294 | 0.021 | 0.004 | 1.40E-06 | 0.003 | 0.008 | 0.749 |
|  |  |  | rs6772 |  | C | 0.267 | -0.021 | 0.005 | 3.70E-06 | 0.033 | 0.009 | 4.00E-04 |
|  |  |  | rs6948971 |  | A | 0.804 | -0.025 | 0.005 | 7.20E-07 | 0.015 | 0.010 | 0.143 |
|  |  |  | rs7329346 |  | C | 0.588 | -0.020 | 0.004 | 1.90E-06 | 0.001 | 0.008 | 0.948 |
|  |  |  | rs7827165 | rs10106327 (0.97) | C | 0.497 | -0.019 | 0.004 | 3.00E-06 | 0.011 | 0.008 | 0.164 |
|  |  |  | rs7932863 | rs7395567 (0.99) | A | 0.269 | 0.021 | 0.005 | 4.40E-06 | 0.003 | 0.010 | 0.754 |
| Chronotype | Caffeine intake | p<5×10^-8^ | rs10157197 |  | G | 0.602 | 0.025 | 0.004 | 1.00E-09 | -0.004 | 0.008 | 0.614 |
|  |  |  | rs12040629 | rs11162296 (1.0) | G | 0.839 | -0.037 | 0.005 | 2.40E-12 | -0.013 | 0.010 | 0.202 |
|  |  |  | rs2168817 | rs12635074 (0.98) | G | 0.681 | -0.023 | 0.004 | 6.90E-08 | -0.002 | 0.008 | 0.763 |
|  |  |  | rs2653349 | rs76899638 (0.99) | A | 0.216 | 0.026 | 0.005 | 5.50E-08 | -0.003 | 0.009 | 0.770 |
|  |  |  | rs4821940 |  | T | 0.447 | 0.022 | 0.004 | 3.40E-08 | -0.002 | 0.008 | 0.768 |
|  |  |  | rs4912138 | rs2050122 (1.0) | A | 0.195 | 0.027 | 0.005 | 4.20E-08 | 0.010 | 0.010 | 0.333 |
|  |  |  | rs516134 |  | C | 0.031 | 0.081 | 0.011 | 8.90E-13 | -0.034 | 0.025 | 0.179 |
|  |  |  | rs9961653 |  | T | 0.422 | 0.023 | 0.004 | 9.60E-09 | -0.005 | 0.008 | 0.555 |
| Chronotype | Caffeine intake | p<1×10^-5^ | rs10113427 |  | A | 0.763 | 0.022 | 0.005 | 1.70E-06 | -0.013 | 0.009 | 0.156 |
|  |  |  | rs10157197 |  | G | 0.602 | 0.025 | 0.004 | 1.00E-09 | -0.004 | 0.008 | 0.614 |
|  |  |  | rs10269368 |  | A | 0.736 | 0.024 | 0.005 | 9.30E-08 | 0.003 | 0.009 | 0.724 |
|  |  |  | rs10864316 | rs7545893 (0.99) | A | 0.804 | -0.025 | 0.005 | 5.70E-07 | -0.006 | 0.010 | 0.524 |
|  |  |  | rs10931166 | rs62198772 (0.91) | C | 0.604 | 0.016 | 0.004 | 4.40E-05 | -0.004 | 0.008 | 0.577 |
|  |  |  | rs11080887 |  | A | 0.867 | -0.026 | 0.006 | 5.50E-06 | -0.002 | 0.011 | 0.832 |
|  |  |  | rs11596752 | rs78682903 (0.95) | G | 0.889 | -0.027 | 0.006 | 1.20E-05 | -0.001 | 0.013 | 0.964 |
|  |  |  | rs11699264 |  | G | 0.847 | 0.026 | 0.006 | 3.10E-06 | 0.008 | 0.012 | 0.482 |
|  |  |  | rs11841507 | rs75049912 (0.82) | C | 0.964 | -0.043 | 0.011 | 4.30E-05 | 0.000 | 0.022 | 0.985 |
|  |  |  | rs11841797 | rs376908252 (0.96) | A | 0.686 | -0.020 | 0.004 | 1.90E-06 | 0.024 | 0.009 | 0.006 |
|  |  |  | rs12022460 | rs72720396 (0.84) | G | 0.746 | -0.023 | 0.005 | 3.60E-07 | -0.006 | 0.009 | 0.519 |
|  |  |  | rs12040629 | rs113240734 (1.0) | G | 0.839 | -0.037 | 0.005 | 2.40E-12 | -0.013 | 0.010 | 0.202 |
|  |  |  | rs12241819 | rs7903778 (0.93) | G | 0.721 | -0.019 | 0.004 | 2.40E-05 | -0.007 | 0.008 | 0.422 |
|  |  |  | rs12580830 |  | C | 0.481 | 0.019 | 0.004 | 1.70E-06 | -0.002 | 0.008 | 0.815 |
|  |  |  | rs12635403 | rs12635074 (0.97) | C | 0.681 | -0.023 | 0.004 | 7.60E-08 | -0.003 | 0.008 | 0.701 |
|  |  |  | rs12651919 | rs12657877 (0.91) | A | 0.840 | -0.022 | 0.005 | 3.20E-05 | -0.004 | 0.011 | 0.718 |
|  |  |  | rs13133212 |  | G | 0.883 | -0.029 | 0.006 | 1.80E-06 | 0.013 | 0.014 | 0.358 |
|  |  |  | rs13290794 | rs17487601 (0.95) | G | 0.630 | 0.018 | 0.004 | 6.80E-06 | 0.011 | 0.008 | 0.158 |
|  |  |  | rs1347531 | rs8055492 (0.99) | C | 0.633 | -0.019 | 0.004 | 3.40E-06 | -0.007 | 0.008 | 0.402 |
|  |  |  | rs1464776 |  | T | 0.481 | 0.019 | 0.004 | 1.90E-06 | 0.002 | 0.008 | 0.770 |
|  |  |  | rs16939130 |  | T | 0.779 | 0.026 | 0.005 | 6.00E-08 | 0.011 | 0.009 | 0.232 |
|  |  |  | rs17311976 |  | T | 0.810 | 0.025 | 0.005 | 8.60E-07 | -0.013 | 0.011 | 0.219 |
|  |  |  | rs17454584 | rs45515895 (0.97) | A | 0.782 | 0.020 | 0.005 | 2.50E-05 | 0.010 | 0.009 | 0.269 |
|  |  |  | rs17659542 |  | C | 0.846 | -0.028 | 0.005 | 2.70E-07 | 0.011 | 0.011 | 0.303 |
|  |  |  | rs1889060 |  | A | 0.297 | -0.022 | 0.004 | 4.00E-07 | -0.006 | 0.009 | 0.471 |
|  |  |  | rs2035366 | rs1383714 (1.0) | G | 0.797 | -0.021 | 0.005 | 1.30E-05 | -0.015 | 0.009 | 0.104 |
|  |  |  | rs2163761 |  | C | 0.707 | 0.021 | 0.004 | 1.60E-06 | 0.020 | 0.009 | 0.019 |
|  |  |  | rs2244661 | rs2578094 (1.0) | G | 0.265 | 0.019 | 0.004 | 1.20E-05 | 0.002 | 0.009 | 0.807 |
|  |  |  | rs238889 |  | T | 0.592 | -0.019 | 0.004 | 1.30E-06 | 0.005 | 0.008 | 0.526 |
|  |  |  | rs3739070 | rs74409360 (0.93) | A | 0.913 | 0.035 | 0.007 | 3.40E-07 | -0.011 | 0.014 | 0.442 |
|  |  |  | rs3852786 |  | C | 0.505 | 0.019 | 0.004 | 9.10E-07 | -0.004 | 0.008 | 0.578 |
|  |  |  | rs3887436 |  | A | 0.578 | 0.020 | 0.004 | 7.60E-07 | 0.010 | 0.008 | 0.195 |
|  |  |  | rs4245555 | rs4245556 (0.94) | T | 0.588 | -0.020 | 0.004 | 1.20E-06 | -0.009 | 0.008 | 0.270 |
|  |  |  | rs4662327 |  | G | 0.630 | 0.020 | 0.004 | 6.50E-07 | 0.018 | 0.008 | 0.023 |
|  |  |  | rs4800617 |  | A | 0.401 | -0.019 | 0.004 | 1.80E-06 | -0.005 | 0.008 | 0.544 |
|  |  |  | rs4821940 |  | T | 0.447 | 0.022 | 0.004 | 3.40E-08 | -0.002 | 0.008 | 0.768 |
|  |  |  | rs4912138 | rs2050122 (1.0) | A | 0.195 | 0.027 | 0.005 | 4.20E-08 | 0.010 | 0.010 | 0.333 |
|  |  |  | rs516016 | rs67019387 (0.85) | A | 0.520 | -0.018 | 0.004 | 8.90E-06 | -0.009 | 0.008 | 0.258 |
|  |  |  | rs516134 |  | C | 0.031 | 0.081 | 0.011 | 8.90E-13 | -0.034 | 0.025 | 0.179 |
|  |  |  | rs595877 |  | G | 0.572 | 0.021 | 0.004 | 1.30E-07 | 0.008 | 0.008 | 0.328 |
|  |  |  | rs6002686 |  | G | 0.482 | -0.019 | 0.004 | 1.80E-06 | 0.001 | 0.008 | 0.903 |
|  |  |  | rs6850095 |  | C | 0.904 | 0.032 | 0.007 | 2.00E-06 | -0.001 | 0.013 | 0.922 |
|  |  |  | rs698820 | rs698814 (0.97) | G | 0.236 | -0.022 | 0.005 | 3.10E-06 | 0.004 | 0.009 | 0.665 |
|  |  |  | rs7081035 | rs111623497 (1.0) | C | 0.974 | 0.059 | 0.012 | 1.60E-06 | -0.008 | 0.028 | 0.770 |
|  |  |  | rs7251052 |  | A | 0.818 | -0.023 | 0.005 | 7.10E-06 | 0.017 | 0.010 | 0.095 |
|  |  |  | rs7297861 | rs73405628 (1.0) | T | 0.901 | 0.033 | 0.007 | 5.90E-07 | 0.004 | 0.013 | 0.743 |
|  |  |  | rs7342459 |  | T | 0.968 | 0.050 | 0.011 | 5.70E-06 | 0.003 | 0.026 | 0.897 |
|  |  |  | rs7492369 | rs55695162 (0.81) | C | 0.749 | -0.020 | 0.005 | 1.40E-05 | -0.010 | 0.010 | 0.303 |
|  |  |  | rs7563917 |  | C | 0.582 | 0.019 | 0.004 | 1.80E-06 | 0.010 | 0.008 | 0.179 |
|  |  |  | rs7711883 |  | C | 0.844 | -0.024 | 0.005 | 7.50E-06 | -0.002 | 0.011 | 0.876 |
|  |  |  | rs7781395 |  | C | 0.548 | 0.018 | 0.004 | 4.50E-06 | 0.003 | 0.008 | 0.706 |
|  |  |  | rs797148 |  | C | 0.917 | -0.033 | 0.007 | 4.80E-06 | 0.017 | 0.013 | 0.193 |
|  |  |  | rs7976870 |  | G | 0.496 | -0.019 | 0.004 | 1.50E-06 | -0.004 | 0.008 | 0.613 |
|  |  |  | rs872956 |  | T | 0.768 | -0.024 | 0.005 | 4.00E-07 | -0.016 | 0.009 | 0.077 |
| Insomnia | Caffeine intake | p<5×10^-8^ | rs11693221 | rs115087496 (0.96) | T | 0.048 | 0.171 | 0.023 | 3.79E-14 | 0.011 | 0.023 | 0.629 |
| Insomnia | Caffeine intake | p<1×10^-5^ | rs10250103 |  | T | 0.479 | 0.047 | 0.010 | 6.26E-07 | 0.011 | 0.008 | 0.155 |
|  |  |  | rs11039701 | rs1994140 (1.0) | G | 0.564 | -0.045 | 0.010 | 1.60E-06 | -0.003 | 0.008 | 0.656 |
|  |  |  | rs11653468 | rs72835405 (1.0) | A | 0.234 | -0.049 | 0.011 | 9.08E-06 | 0.006 | 0.010 | 0.543 |
|  |  |  | rs1547668 |  | G | 0.796 | -0.054 | 0.012 | 3.61E-06 | 0.004 | 0.010 | 0.708 |
|  |  |  | rs16966956 | rs4772694 (1.0) | G | 0.281 | -0.047 | 0.011 | 8.66E-06 | 0.002 | 0.009 | 0.847 |
|  |  |  | rs17191759 | rs145265872 (0.80) | C | 0.042 | -0.107 | 0.023 | 4.43E-06 | 0.011 | 0.020 | 0.568 |
|  |  |  | rs17400178 |  | T | 0.071 | 0.085 | 0.019 | 6.63E-06 | -0.010 | 0.017 | 0.574 |
|  |  |  | rs17482814 | rs72664496 (0.88) | C | 0.070 | -0.062 | 0.018 | 8.17E-04 | 0.052 | 0.019 | 0.006 |
|  |  |  | rs2087000 |  | C | 0.223 | 0.051 | 0.011 | 7.95E-06 | 0.001 | 0.009 | 0.910 |
|  |  |  | rs208827 |  | G | 0.636 | 0.051 | 0.010 | 2.24E-07 | -0.011 | 0.008 | 0.194 |
|  |  |  | rs2219778 | rs149326099 (0.88 | T | 0.039 | -0.105 | 0.024 | 1.54E-05 | 0.004 | 0.019 | 0.822 |
|  |  |  | rs2673609 |  | G | 0.360 | 0.045 | 0.010 | 4.98E-06 | -0.001 | 0.008 | 0.956 |
|  |  |  | rs5752673 | rs738475 (0.83) | T | 0.242 | 0.049 | 0.011 | 7.78E-06 | 0.001 | 0.009 | 0.943 |
|  |  |  | rs641783 | rs865199 (0.96) | A | 0.065 | -0.086 | 0.019 | 7.36E-06 | -0.005 | 0.015 | 0.760 |
|  |  |  | rs7332332 |  | G | 0.497 | -0.044 | 0.009 | 3.52E-06 | 0.008 | 0.008 | 0.277 |
|  |  |  | rs7423933 |  | A | 0.735 | -0.047 | 0.011 | 9.05E-06 | 0.010 | 0.009 | 0.259 |
| Plasma caffeine | Sleep duration | p<5×10^-8^ | rs6968554 |  | A | 0.366 | 0.151 | 0.021 | 5.36E-13 | 0.004 | 0.004 | 0.290 |
| Plasma caffeine | Sleep duration | p<1×10^-5^ | rs10088336 |  | A | 0.773 | -0.115 | 0.025 | 4.80E-06 | 0.001 | 0.005 | 0.840 |
|  |  |  | rs10493487 |  | A | 0.976 | 0.310 | 0.069 | 6.20E-06 | -0.021 | 0.013 | 0.092 |
|  |  |  | rs10516471 |  | A | 0.607 | -0.093 | 0.021 | 7.09E-06 | -0.001 | 0.004 | 0.720 |
|  |  |  | rs11622417 | rs11157465 (0.97) | T | 0.551 | 0.096 | 0.021 | 4.97E-06 | 0.001 | 0.004 | 0.850 |
|  |  |  | rs12566116 |  | A | 0.085 | 0.184 | 0.038 | 1.07E-06 | 0.003 | 0.007 | 0.700 |
|  |  |  | rs17328249 |  | A | 0.446 | 0.097 | 0.021 | 4.98E-06 | -0.006 | 0.004 | 0.110 |
|  |  |  | rs1992145 |  | A | 0.572 | -0.109 | 0.021 | 3.11E-07 | -0.002 | 0.004 | 0.690 |
|  |  |  | rs2270969 |  | A | 0.971 | -0.278 | 0.063 | 8.78E-06 | 0.015 | 0.012 | 0.210 |
|  |  |  | rs3764715 |  | A | 0.341 | -0.111 | 0.022 | 6.10E-07 | -0.003 | 0.004 | 0.470 |
|  |  |  | rs4239278 |  | T | 0.285 | -0.106 | 0.023 | 5.55E-06 | 0.003 | 0.004 | 0.500 |
|  |  |  | rs6968554 |  | A | 0.366 | 0.151 | 0.021 | 5.36E-13 | 0.004 | 0.004 | 0.290 |
| Plasma caffeine | Chronotype | p<5×10^-8^ | rs6968554 |  | A | 0.366 | 0.151 | 0.021 | 5.36E-13 | -0.008 | 0.004 | 0.045 |
| Plasma caffeine | Chronotype | p<1×10^-5^ | rs10088336 |  | A | 0.773 | -0.115 | 0.025 | 4.80E-06 | 0.008 | 0.005 | 0.072 |
|  |  |  | rs10493487 |  | A | 0.976 | 0.310 | 0.069 | 6.20E-06 | 0.006 | 0.012 | 0.640 |
|  |  |  | rs10516471 |  | A | 0.607 | -0.093 | 0.021 | 7.09E-06 | -0.004 | 0.004 | 0.340 |
|  |  |  | rs11622417 | rs11157465 (0.97) | T | 0.551 | 0.096 | 0.021 | 4.97E-06 | -0.005 | 0.004 | 0.170 |
|  |  |  | rs12566116 |  | A | 0.085 | 0.184 | 0.038 | 1.07E-06 | 0.002 | 0.007 | 0.800 |
|  |  |  | rs17328249 |  | A | 0.446 | 0.097 | 0.021 | 4.98E-06 | -0.002 | 0.004 | 0.550 |
|  |  |  | rs1992145 |  | A | 0.572 | -0.109 | 0.021 | 3.11E-07 | 0.007 | 0.004 | 0.076 |
|  |  |  | rs2270969 |  | A | 0.971 | -0.278 | 0.063 | 8.78E-06 | 0.034 | 0.012 | 0.006 |
|  |  |  | rs3764715 |  | A | 0.341 | -0.111 | 0.022 | 6.10E-07 | 0.000 | 0.004 | 0.940 |
|  |  |  | rs4239278 |  | T | 0.285 | -0.106 | 0.023 | 5.55E-06 | 0.003 | 0.004 | 0.560 |
|  |  |  | rs6968554 |  | A | 0.366 | 0.151 | 0.021 | 5.36E-13 | -0.008 | 0.004 | 0.045 |
| Plasma caffeine | Insomnia | p<5×10^-8^ | rs6968554 |  | A | 0.366 | 0.151 | 0.021 | 5.36E-13 | 0.003 | 0.010 | 0.770 |
| Plasma caffeine | Insomnia | p<1×10^-5^ | rs10088336 |  | A | 0.773 | -0.115 | 0.025 | 4.80E-06 | -0.007 | 0.011 | 0.512 |
|  |  |  | rs10493487 |  | A | 0.976 | 0.310 | 0.069 | 6.20E-06 | 0.049 | 0.030 | 0.099 |
|  |  |  | rs10516471 |  | A | 0.607 | -0.093 | 0.021 | 7.09E-06 | -0.010 | 0.010 | 0.282 |
|  |  |  | rs11157465 |  | T | 0.551 | 0.096 | 0.021 | 4.97E-06 | 0.012 | 0.009 | 0.174 |
|  |  |  | rs12566116 |  | A | 0.085 | 0.184 | 0.038 | 1.07E-06 | -0.024 | 0.017 | 0.139 |
|  |  |  | rs17328249 |  | A | 0.446 | 0.097 | 0.021 | 4.98E-06 | -0.007 | 0.009 | 0.461 |
|  |  |  | rs1992145 |  | A | 0.572 | -0.109 | 0.021 | 3.11E-07 | 0.018 | 0.010 | 0.058 |
|  |  |  | rs2270969 |  | A | 0.971 | -0.278 | 0.063 | 8.78E-06 | -0.027 | 0.030 | 0.355 |
|  |  |  | rs3764715 |  | A | 0.341 | -0.111 | 0.022 | 6.10E-07 | -0.002 | 0.010 | 0.836 |
|  |  |  | rs4239278 |  | T | 0.285 | -0.106 | 0.023 | 5.55E-06 | 0.000 | 0.010 | 0.982 |
|  |  |  | rs6968554 |  | A | 0.366 | 0.151 | 0.021 | 5.36E-13 | 0.003 | 0.010 | 0.770 |
| Sleep duration | Plasma caffeine | p<5×10^-8^ | rs17269154 | rs17190618 (0.96) | C | 0.843 | -0.031 | 0.005 | 1.00E-08 | -0.018 | 0.029 | 0.527 |
|  |  |  | rs1823125 | rs1807282 (0.95) | A | 0.781 | -0.038 | 0.005 | 1.90E-15 | -0.003 | 0.025 | 0.917 |
| Sleep duration | Plasma caffeine | p<1×10^-5^ | rs10799034 | rs6425885 (0.97) | T | 0.293 | 0.020 | 0.004 | 2.50E-06 | 0.011 | 0.023 | 0.643 |
|  |  |  | rs11042154 | rs10840160 (0.84) | G | 0.547 | 0.018 | 0.004 | 1.50E-05 | 0.005 | 0.021 | 0.826 |
|  |  |  | rs11964802 | rs67059016 (1.0) | A | 0.712 | 0.019 | 0.004 | 8.50E-06 | 0.011 | 0.023 | 0.637 |
|  |  |  | rs154917 | rs62099775 (1.0) | C | 0.681 | -0.019 | 0.004 | 7.40E-06 | -0.025 | 0.023 | 0.277 |
|  |  |  | rs17121264 |  | A | 0.926 | 0.036 | 0.008 | 5.00E-06 | -0.013 | 0.041 | 0.743 |
|  |  |  | rs17269154 | rs17190618 (0.96) | C | 0.843 | -0.031 | 0.005 | 1.00E-08 | -0.018 | 0.029 | 0.527 |
|  |  |  | rs1823125 | rs62158211 (0.95) | A | 0.781 | -0.038 | 0.005 | 1.90E-15 | -0.003 | 0.025 | 0.917 |
|  |  |  | rs2846581 |  | C | 0.275 | -0.024 | 0.004 | 1.20E-07 | -0.020 | 0.023 | 0.389 |
|  |  |  | rs342742 | rs342745 (0.99) | A | 0.202 | -0.022 | 0.005 | 1.10E-05 | -0.040 | 0.026 | 0.126 |
|  |  |  | rs6466057 | rs7797160 (0.88) | T | 0.337 | 0.015 | 0.004 | 2.70E-04 | -0.029 | 0.022 | 0.190 |
|  |  |  | rs6772 |  | C | 0.267 | -0.021 | 0.004 | 3.70E-06 | -0.024 | 0.024 | 0.318 |
|  |  |  | rs7073430 | rs7895364 (0.93) | C | 0.588 | 0.019 | 0.004 | 1.70E-06 | -0.016 | 0.021 | 0.452 |
|  |  |  | rs7329346 |  | C | 0.588 | -0.020 | 0.004 | 1.90E-06 | 0.014 | 0.021 | 0.503 |
|  |  |  | rs7395835 | rs7395567 (1.0) | C | 0.311 | 0.019 | 0.004 | 2.80E-05 | 0.006 | 0.023 | 0.780 |
|  |  |  | rs7575253 | rs35684737 (0.93) | C | 0.867 | -0.026 | 0.006 | 7.20E-06 | -0.040 | 0.030 | 0.191 |
|  |  |  | rs811919 | rs3095508 (0.99) | G | 0.592 | 0.018 | 0.004 | 4.50E-06 | 0.003 | 0.022 | 0.899 |
| Chronotype | Plasma caffeine | p<5×10^-8^ | rs11582254 | rs4912138 (0.84) | T | 0.194 | 0.025 | 0.005 | 4.80E-07 | -0.011 | 0.027 | 0.698 |
|  |  |  | rs1421622 | rs1075265 (0.90) | A | 0.481 | -0.023 | 0.004 | 1.00E-08 | -0.002 | 0.020 | 0.933 |
|  |  |  | rs1694379 | rs10157197 (0.93) | C | 0.387 | -0.023 | 0.004 | 6.50E-09 | -0.020 | 0.022 | 0.350 |
|  |  |  | rs3816575 | rs2168817 (0.80) | G | 0.680 | -0.021 | 0.004 | 4.00E-07 | 0.012 | 0.022 | 0.584 |
| Chronotype | Plasma caffeine | p<1×10^-5^ | rs10035367 | rs7711883 (0.99) | C | 0.845 | -0.023 | 0.005 | 1.80E-05 | 0.005 | 0.029 | 0.852 |
|  |  |  | rs10092128 | rs16939130 (1.0) | C | 0.779 | 0.025 | 0.005 | 7.30E-08 | -0.016 | 0.026 | 0.534 |
|  |  |  | rs10108049 | rs10113427 (0.97) | G | 0.763 | 0.022 | 0.005 | 2.20E-06 | -0.029 | 0.024 | 0.240 |
|  |  |  | rs10233473 | rs10269368 (0.93) | C | 0.748 | 0.023 | 0.005 | 3.60E-07 | -0.023 | 0.025 | 0.345 |
|  |  |  | rs10513934 | rs11080887 (1.0) | T | 0.867 | -0.026 | 0.006 | 5.80E-06 | 0.029 | 0.031 | 0.345 |
|  |  |  | rs10880855 | rs7976870 (0.99) | C | 0.495 | -0.019 | 0.004 | 2.20E-06 | -0.001 | 0.021 | 0.980 |
|  |  |  | rs11075924 | rs3852786 (0.95) | C | 0.509 | 0.019 | 0.004 | 1.50E-06 | 0.007 | 0.021 | 0.752 |
|  |  |  | rs11582254 | rs2050122 (0.84) | T | 0.194 | 0.025 | 0.005 | 4.80E-07 | -0.011 | 0.027 | 0.698 |
|  |  |  | rs11699264 |  | G | 0.847 | 0.026 | 0.005 | 3.10E-06 | 0.020 | 0.029 | 0.502 |
|  |  |  | rs12022460 | rs72720396 (0.84) | G | 0.746 | -0.023 | 0.005 | 3.60E-07 | 0.040 | 0.024 | 0.103 |
|  |  |  | rs12241819 | rs7903778 (0.93) | G | 0.721 | -0.018 | 0.004 | 2.40E-05 | 0.020 | 0.023 | 0.395 |
|  |  |  | rs12580830 |  | C | 0.481 | 0.019 | 0.004 | 1.70E-06 | -0.002 | 0.021 | 0.917 |
|  |  |  | rs12656140 | rs12657877 (0.91) | C | 0.840 | -0.022 | 0.005 | 2.90E-05 | 0.036 | 0.029 | 0.220 |
|  |  |  | rs13290794 | rs17487601 (0.95) | G | 0.629 | 0.018 | 0.004 | 6.80E-06 | -0.032 | 0.022 | 0.147 |
|  |  |  | rs1421622 | rs1075265 (0.90) | A | 0.481 | -0.023 | 0.004 | 1.00E-08 | -0.002 | 0.020 | 0.933 |
|  |  |  | rs1446710 | rs7563917 (0.96) | G | 0.578 | 0.018 | 0.004 | 9.20E-06 | 0.046 | 0.021 | 0.028 |
|  |  |  | rs1694379 | rs10157197 (0.93) | C | 0.387 | -0.023 | 0.004 | 6.50E-09 | -0.020 | 0.022 | 0.350 |
|  |  |  | rs16950293 | rs73405628 (0.94) | G | 0.893 | 0.029 | 0.006 | 3.40E-06 | -0.034 | 0.032 | 0.293 |
|  |  |  | rs17374439 | rs7545893 (0.99) | C | 0.804 | -0.025 | 0.005 | 5.40E-07 | 0.018 | 0.026 | 0.489 |
|  |  |  | rs17454584 | rs45515895 (0.97) | A | 0.782 | 0.020 | 0.005 | 2.50E-05 | 0.020 | 0.025 | 0.407 |
|  |  |  | rs17822102 | rs8055492 (0.83) | A | 0.655 | -0.016 | 0.004 | 1.30E-04 | -0.003 | 0.022 | 0.875 |
|  |  |  | rs1992292 | rs7781395 (0.99) | T | 0.544 | 0.018 | 0.004 | 5.10E-06 | 0.009 | 0.021 | 0.661 |
|  |  |  | rs2019834 | rs1383714 (1.0) | G | 0.798 | -0.021 | 0.005 | 1.20E-05 | 0.025 | 0.026 | 0.327 |
|  |  |  | rs2255696 | rs2578094 (1.0) | T | 0.266 | 0.019 | 0.004 | 1.40E-05 | 0.000 | 0.024 | 0.986 |
|  |  |  | rs238889 |  | T | 0.591 | -0.019 | 0.004 | 1.30E-06 | -0.001 | 0.022 | 0.977 |
|  |  |  | rs3816575 | rs12635074 (0.81) | G | 0.680 | -0.021 | 0.004 | 4.00E-07 | 0.012 | 0.022 | 0.584 |
|  |  |  | rs4245555 | rs4245556 (0.94) | T | 0.588 | -0.020 | 0.004 | 1.20E-06 | -0.032 | 0.020 | 0.117 |
|  |  |  | rs4447605 | rs6729830 (0.95) | A | 0.438 | 0.018 | 0.004 | 3.20E-06 | 0.013 | 0.021 | 0.533 |
|  |  |  | rs4600609 | rs62198772 (0.91) | G | 0.604 | 0.017 | 0.004 | 3.60E-05 | 0.023 | 0.022 | 0.276 |
|  |  |  | rs4662327 |  | G | 0.630 | 0.020 | 0.004 | 6.50E-07 | -0.016 | 0.022 | 0.466 |
|  |  |  | rs4800617 |  | A | 0.401 | -0.019 | 0.004 | 1.80E-06 | 0.008 | 0.021 | 0.711 |
|  |  |  | rs4821940 |  | T | 0.447 | 0.022 | 0.004 | 3.40E-08 | -0.010 | 0.021 | 0.631 |
|  |  |  | rs516134 |  | C | 0.031 | 0.081 | 0.011 | 8.90E-13 | 0.064 | 0.065 | 0.327 |
|  |  |  | rs595877 |  | G | 0.572 | 0.021 | 0.004 | 1.30E-07 | 0.004 | 0.021 | 0.851 |
|  |  |  | rs6002686 |  | G | 0.482 | -0.019 | 0.004 | 1.80E-06 | 0.018 | 0.021 | 0.391 |
|  |  |  | rs6850095 |  | C | 0.904 | 0.032 | 0.007 | 2.00E-06 | 0.033 | 0.035 | 0.347 |
|  |  |  | rs6971007 | rs1464776 (0.86) | C | 0.438 | 0.018 | 0.004 | 4.90E-06 | -0.026 | 0.021 | 0.224 |
|  |  |  | rs698791 | rs698814 (0.94) | A | 0.241 | -0.020 | 0.005 | 2.10E-05 | 0.023 | 0.024 | 0.322 |
|  |  |  | rs7251052 |  | A | 0.818 | -0.023 | 0.005 | 7.10E-06 | 0.019 | 0.028 | 0.501 |
|  |  |  | rs7342459 |  | T | 0.968 | 0.050 | 0.011 | 5.70E-06 | 0.031 | 0.064 | 0.625 |
|  |  |  | rs7492369 | rs55695162 (0.81) | C | 0.749 | -0.020 | 0.005 | 1.40E-05 | 0.053 | 0.024 | 0.030 |
|  |  |  | rs797148 |  | C | 0.917 | -0.033 | 0.007 | 4.80E-06 | 0.013 | 0.038 | 0.732 |
| Insomnia | Plasma caffeine | p<5×10^-8^ | rs11693221 |  | T | 0.048 | 0.171 | 0.023 | 3.79E-14 | 0.080 | 0.048 | 0.097 |
| Insomnia | Plasma caffeine | p<1×10^-5^ | rs11039701 | rs1994140 (1.0) | G | 0.564 | -0.045 | 0.009 | 1.60E-06 | -0.013 | 0.021 | 0.529 |
|  |  |  | rs11653468 | rs72835405 (0.99) | A | 0.233 | -0.049 | 0.011 | 9.08E-06 | -0.014 | 0.024 | 0.568 |
|  |  |  | rs12705971 | rs10250103 (0.98) | T | 0.475 | 0.046 | 0.009 | 1.48E-06 | -0.030 | 0.020 | 0.134 |
|  |  |  | rs1372256 | rs2087000 (0.83) | T | 0.203 | 0.049 | 0.012 | 2.49E-05 | 0.034 | 0.026 | 0.190 |
|  |  |  | rs1530399 | rs7332332 (0.85) | T | 0.470 | -0.042 | 0.009 | 8.15E-06 | 0.015 | 0.021 | 0.477 |
|  |  |  | rs1547668 |  | G | 0.796 | -0.054 | 0.012 | 3.61E-06 | 0.010 | 0.025 | 0.698 |
|  |  |  | rs16966956 | rs4772694 (1.0) | G | 0.281 | -0.047 | 0.010 | 8.66E-06 | -0.006 | 0.024 | 0.812 |
|  |  |  | rs17400178 |  | T | 0.071 | 0.084 | 0.019 | 6.63E-06 | -0.047 | 0.039 | 0.232 |
|  |  |  | rs2219778 | rs149326099 (0.88) | T | 0.039 | -0.105 | 0.024 | 1.54E-05 | -0.061 | 0.052 | 0.246 |
|  |  |  | rs2673608 | rs2673609 (0.96) | C | 0.346 | 0.043 | 0.010 | 1.37E-05 | 0.018 | 0.022 | 0.418 |
|  |  |  | rs3913801 | rs6437732 (0.86) | A | 0.484 | -0.036 | 0.009 | 1.61E-04 | 0.014 | 0.021 | 0.512 |
|  |  |  | rs5752673 | rs738475 (0.83) | T | 0.242 | 0.049 | 0.011 | 7.78E-06 | 0.009 | 0.024 | 0.721 |
|  |  |  | rs641783 | rs865199 (0.96) | A | 0.065 | -0.086 | 0.019 | 7.36E-06 | -0.041 | 0.042 | 0.329 |
|  |  |  | rs9383763 | rs9383762 (0.95) | C | 0.478 | 0.037 | 0.009 | 7.97E-05 | 0.014 | 0.021 | 0.496 |
| Caffeine metabolic rate | Sleep duration | p<5×10^-8^ | rs6968554 |  | A | 0.375 | -0.222 | 0.026 | 3.22E-17 | 0.004 | 0.004 | 0.290 |
|  |  |  |  |  |  |  |  |  |  |  |  |  |
| Caffeine metabolic rate | Sleep duration | p<1×10^-5^ | rs10973772 |  | T | 0.396 | 0.124 | 0.028 | 9.15E-06 | -0.006 | 0.004 | 0.140 |
|  |  |  | rs1410138 |  | T | 0.976 | -0.445 | 0.090 | 6.59E-07 | -0.002 | 0.012 | 0.870 |
|  |  |  | rs2828656 |  | T | 0.344 | 0.144 | 0.029 | 5.81E-07 | -0.004 | 0.005 | 0.380 |
|  |  |  | rs6665339 |  | A | 0.280 | -0.145 | 0.031 | 2.04E-06 | -0.002 | 0.004 | 0.580 |
|  |  |  | rs6958133 |  | A | 0.020 | 0.434 | 0.098 | 9.22E-06 | -0.005 | 0.013 | 0.700 |
|  |  |  | rs6968554 |  | A | 0.375 | -0.222 | 0.026 | 3.22E-17 | 0.004 | 0.004 | 0.290 |
|  |  |  | rs726576 |  | T | 0.652 | -0.131 | 0.029 | 5.64E-06 | 0.007 | 0.004 | 0.130 |
|  |  |  | rs780093 |  | T | 0.400 | -0.117 | 0.026 | 6.85E-06 | 0.005 | 0.004 | 0.190 |
| Caffeine metabolic rate | Chronotype | p<5×10^-8^ | rs6968554 |  | A | 0.375 | -0.222 | 0.026 | 3.22E-17 | -0.008 | 0.004 | 0.045 |
|  |  |  |  |  |  |  |  |  |  |  |  |  |
| Caffeine metabolic rate | Chronotype | p<1×10^-5^ | rs10973772 |  | T | 0.396 | 0.124 | 0.028 | 9.15E-06 | -0.004 | 0.004 | 0.300 |
|  |  |  | rs1410138 |  | T | 0.976 | -0.445 | 0.090 | 6.59E-07 | 0.013 | 0.012 | 0.270 |
|  |  |  | rs2828656 |  | T | 0.344 | 0.144 | 0.029 | 5.81E-07 | -0.006 | 0.004 | 0.180 |
|  |  |  | rs6665339 |  | A | 0.280 | -0.145 | 0.031 | 2.04E-06 | 0.001 | 0.004 | 0.860 |
|  |  |  | rs6958133 |  | A | 0.020 | 0.434 | 0.098 | 9.22E-06 | 0.010 | 0.013 | 0.420 |
|  |  |  | rs6968554 |  | A | 0.375 | -0.222 | 0.026 | 3.22E-17 | -0.008 | 0.004 | 0.045 |
|  |  |  | rs726576 |  | T | 0.652 | -0.131 | 0.029 | 5.64E-06 | -0.005 | 0.004 | 0.250 |
|  |  |  | rs780093 |  | T | 0.400 | -0.117 | 0.026 | 6.85E-06 | -0.004 | 0.004 | 0.380 |
| Caffeine metabolic rate | Insomnia | p<5×10^-8^ | rs6968554 |  | A | 0.375 | -0.222 | 0.026 | 3.22E-17 | 0.003 | 0.010 | 0.770 |
|  |  |  | rs8102683 |  | T | 0.247 | 0.196 | 0.032 | 6.79E-10 | 0.010 | 0.010 | 0.349 |
| Caffeine metabolic rate | Insomnia | p<1×10^-5^ | rs10973772 |  | T | 0.396 | 0.124 | 0.028 | 9.15E-06 | -0.004 | 0.010 | 0.695 |
|  |  |  | rs1410138 |  | T | 0.976 | -0.445 | 0.090 | 6.59E-07 | -0.063 | 0.029 | 0.031 |
|  |  |  | rs2828656 |  | T | 0.344 | 0.144 | 0.029 | 5.81E-07 | 0.012 | 0.010 | 0.268 |
|  |  |  | rs6665339 |  | A | 0.280 | -0.145 | 0.031 | 2.04E-06 | 0.005 | 0.010 | 0.622 |
|  |  |  | rs6958133 |  | A | 0.020 | 0.434 | 0.098 | 9.22E-06 | 0.053 | 0.031 | 0.086 |
|  |  |  | rs6968554 |  | A | 0.375 | -0.222 | 0.026 | 3.22E-17 | 0.003 | 0.010 | 0.770 |
|  |  |  | rs726576 |  | T | 0.652 | -0.131 | 0.029 | 5.64E-06 | -0.015 | 0.010 | 0.131 |
|  |  |  | rs780093 |  | T | 0.400 | -0.117 | 0.026 | 6.85E-06 | 0.000 | 0.010 | 0.977 |
|  |  |  | rs8102683 |  | T | 0.247 | 0.196 | 0.032 | 6.79E-10 | 0.010 | 0.010 | 0.349 |
| Sleep duration | Caffeine metabolic rate | p<5×10^-8^ | rs17269154 | rs17190618 (0.96) | C | 0.843 | -0.031 | 0.005 | 1.00E-08 | -0.022 | 0.038 | 0.554 |
|  |  |  | rs1823125 | rs1807282 (0.95) | A | 0.781 | -0.038 | 0.005 | 1.90E-15 | 0.016 | 0.033 | 0.620 |
| Sleep duration | Caffeine metabolic rate | p<1×10^-5^ | rs10799034 | rs6425885 (0.97) | T | 0.293 | 0.020 | 0.004 | 2.50E-06 | 0.035 | 0.030 | 0.242 |
|  |  |  | rs11042154 | rs10840160 (0.84) | G | 0.547 | 0.018 | 0.004 | 1.50E-05 | 0.026 | 0.028 | 0.350 |
|  |  |  | rs11964802 | rs67059016 (1.0) | A | 0.712 | 0.019 | 0.004 | 8.50E-06 | 0.004 | 0.030 | 0.885 |
|  |  |  | rs154917 | rs62099775 (1.0) | C | 0.681 | -0.019 | 0.004 | 7.40E-06 | 0.028 | 0.030 | 0.353 |
|  |  |  | rs17121264 |  | A | 0.926 | 0.036 | 0.008 | 5.00E-06 | 0.014 | 0.051 | 0.790 |
|  |  |  | rs17269154 | rs17190618 (0.96) | C | 0.843 | -0.031 | 0.005 | 1.00E-08 | -0.022 | 0.038 | 0.554 |
|  |  |  | rs1823125 | rs62158211 (0.95) | A | 0.781 | -0.038 | 0.005 | 1.90E-15 | 0.016 | 0.033 | 0.620 |
|  |  |  | rs2846581 |  | C | 0.275 | -0.024 | 0.004 | 1.20E-07 | 0.048 | 0.030 | 0.112 |
|  |  |  | rs342742 | rs342745 (0.99) | A | 0.202 | -0.022 | 0.005 | 1.10E-05 | 0.011 | 0.034 | 0.754 |
|  |  |  | rs6466057 | rs7797160 (0.88) | T | 0.337 | 0.015 | 0.004 | 2.70E-04 | 0.029 | 0.029 | 0.312 |
|  |  |  | rs6772 |  | C | 0.267 | -0.021 | 0.004 | 3.70E-06 | -0.032 | 0.031 | 0.298 |
|  |  |  | rs7073430 | rs7895364 (0.93) | C | 0.588 | 0.019 | 0.004 | 1.70E-06 | -0.058 | 0.028 | 0.036 |
|  |  |  | rs7329346 |  | C | 0.588 | -0.020 | 0.004 | 1.90E-06 | -0.026 | 0.028 | 0.346 |
|  |  |  | rs7395835 | rs7395567 (1.0) | C | 0.311 | 0.019 | 0.004 | 2.80E-05 | -0.015 | 0.030 | 0.620 |
|  |  |  | rs7575253 | rs35684737 (0.93) | C | 0.867 | -0.026 | 0.006 | 7.20E-06 | 0.025 | 0.039 | 0.517 |
|  |  |  | rs811919 | rs3095508 (0.99) | G | 0.592 | 0.018 | 0.004 | 4.50E-06 | -0.029 | 0.028 | 0.306 |
| Chronotype | Caffeine metabolic rate | p<5×10^-8^ | rs11582254 | rs4912138 (0.84) | T | 0.194 | 0.025 | 0.005 | 4.80E-07 | -0.002 | 0.035 | 0.952 |
|  |  |  | rs1421622 | rs1075265 (0.90) | A | 0.481 | -0.023 | 0.004 | 1.00E-08 | -0.030 | 0.026 | 0.243 |
|  |  |  | rs1694379 | rs10157197 (0.93) | C | 0.387 | -0.023 | 0.004 | 6.50E-09 | 0.018 | 0.028 | 0.522 |
|  |  |  | rs3816575 | rs2168817 (0.80) | G | 0.680 | -0.021 | 0.004 | 4.00E-07 | -0.007 | 0.029 | 0.802 |
| Chronotype | Caffeine metabolic rate | p<1×10^-5^ | rs10035367 | rs7711883 (0.99) | C | 0.845 | -0.023 | 0.005 | 1.80E-05 | 0.041 | 0.038 | 0.288 |
|  |  |  | rs10092128 | rs16939130 (1.0) | C | 0.779 | 0.025 | 0.005 | 7.30E-08 | 0.003 | 0.033 | 0.922 |
|  |  |  | rs10108049 | rs10113427 (0.97) | G | 0.763 | 0.022 | 0.005 | 2.20E-06 | -0.020 | 0.032 | 0.534 |
|  |  |  | rs10233473 | rs10269368 (0.93) | C | 0.748 | 0.023 | 0.005 | 3.60E-07 | 0.009 | 0.032 | 0.772 |
|  |  |  | rs10513934 | rs11080887 (1.0) | T | 0.867 | -0.026 | 0.006 | 5.80E-06 | -0.057 | 0.040 | 0.158 |
|  |  |  | rs10880855 | rs7976870 (0.99) | C | 0.495 | -0.019 | 0.004 | 2.20E-06 | -0.032 | 0.027 | 0.243 |
|  |  |  | rs11075924 | rs3852786 (0.95) | C | 0.509 | 0.019 | 0.004 | 1.50E-06 | 0.014 | 0.027 | 0.610 |
|  |  |  | rs11582254 | rs2050122 (0.84) | T | 0.194 | 0.025 | 0.005 | 4.80E-07 | -0.002 | 0.035 | 0.952 |
|  |  |  | rs11699264 |  | G | 0.847 | 0.026 | 0.005 | 3.10E-06 | -0.024 | 0.039 | 0.532 |
|  |  |  | rs12022460 | rs72720396 (0.84) | G | 0.746 | -0.023 | 0.005 | 3.60E-07 | -0.030 | 0.032 | 0.337 |
|  |  |  | rs12241819 | rs7903778 (0.93) | G | 0.721 | -0.018 | 0.004 | 2.40E-05 | -0.009 | 0.031 | 0.780 |
|  |  |  | rs12580830 |  | C | 0.481 | 0.019 | 0.004 | 1.70E-06 | -0.008 | 0.027 | 0.758 |
|  |  |  | rs12656140 | rs12657877 (0.91) | C | 0.840 | -0.022 | 0.005 | 2.90E-05 | -0.023 | 0.038 | 0.553 |
|  |  |  | rs13290794 | rs17487601 (0.95) | G | 0.629 | 0.018 | 0.004 | 6.80E-06 | 0.049 | 0.028 | 0.084 |
|  |  |  | rs1421622 | rs1075265 (0.90) | A | 0.481 | -0.023 | 0.004 | 1.00E-08 | -0.030 | 0.026 | 0.243 |
|  |  |  | rs1446710 | rs7563917 (0.96) | G | 0.578 | 0.018 | 0.004 | 9.20E-06 | -0.013 | 0.028 | 0.629 |
|  |  |  | rs1694379 | rs10157197 (0.93) | C | 0.387 | -0.023 | 0.004 | 6.50E-09 | 0.018 | 0.028 | 0.522 |
|  |  |  | rs16950293 | rs73405628 (0.94) | G | 0.893 | 0.029 | 0.006 | 3.40E-06 | 0.025 | 0.044 | 0.572 |
|  |  |  | rs17374439 | rs7545893 (0.99) | C | 0.804 | -0.025 | 0.005 | 5.40E-07 | -0.008 | 0.035 | 0.814 |
|  |  |  | rs17454584 | rs45515895 (0.97) | A | 0.782 | 0.020 | 0.005 | 2.50E-05 | -0.062 | 0.033 | 0.060 |
|  |  |  | rs17822102 | rs8055492 (0.83) | A | 0.655 | -0.016 | 0.004 | 1.30E-04 | -0.020 | 0.029 | 0.482 |
|  |  |  | rs1992292 | rs7781395 (0.99) | T | 0.544 | 0.018 | 0.004 | 5.10E-06 | -0.065 | 0.027 | 0.017 |
|  |  |  | rs2019834 | rs1383714 (1.0) | G | 0.798 | -0.021 | 0.005 | 1.20E-05 | 0.011 | 0.034 | 0.739 |
|  |  |  | rs2255696 | rs2578094 (1.0) | T | 0.266 | 0.019 | 0.004 | 1.40E-05 | -0.067 | 0.031 | 0.030 |
|  |  |  | rs238889 |  | T | 0.591 | -0.019 | 0.004 | 1.30E-06 | 0.018 | 0.028 | 0.513 |
|  |  |  | rs3816575 | rs12635074 (0.81) | G | 0.680 | -0.021 | 0.004 | 4.00E-07 | -0.007 | 0.029 | 0.802 |
|  |  |  | rs4245555 | rs4245556 (0.94) | T | 0.588 | -0.020 | 0.004 | 1.20E-06 | 0.018 | 0.026 | 0.495 |
|  |  |  | rs4447605 | rs6729830 (0.95) | A | 0.438 | 0.018 | 0.004 | 3.20E-06 | -0.040 | 0.028 | 0.149 |
|  |  |  | rs4600609 | rs62198772 (0.91) | G | 0.604 | 0.017 | 0.004 | 3.60E-05 | -0.024 | 0.028 | 0.387 |
|  |  |  | rs4662327 |  | G | 0.630 | 0.020 | 0.004 | 6.50E-07 | 0.042 | 0.028 | 0.134 |
|  |  |  | rs4800617 |  | A | 0.401 | -0.019 | 0.004 | 1.80E-06 | -0.040 | 0.028 | 0.150 |
|  |  |  | rs4821940 |  | T | 0.447 | 0.022 | 0.004 | 3.40E-08 | 0.046 | 0.028 | 0.098 |
|  |  |  | rs516134 |  | C | 0.031 | 0.081 | 0.011 | 8.90E-13 | -0.002 | 0.083 | 0.984 |
|  |  |  | rs595877 |  | G | 0.572 | 0.021 | 0.004 | 1.30E-07 | 0.029 | 0.028 | 0.295 |
|  |  |  | rs6002686 |  | G | 0.482 | -0.019 | 0.004 | 1.80E-06 | -0.035 | 0.027 | 0.199 |
|  |  |  | rs6850095 |  | C | 0.904 | 0.032 | 0.007 | 2.00E-06 | -0.043 | 0.046 | 0.349 |
|  |  |  | rs6971007 | rs1464776 (0.86) | C | 0.438 | 0.018 | 0.004 | 4.90E-06 | 0.012 | 0.028 | 0.664 |
|  |  |  | rs698791 | rs698814 (0.94) | A | 0.241 | -0.020 | 0.005 | 2.10E-05 | -0.024 | 0.030 | 0.433 |
|  |  |  | rs7251052 |  | A | 0.818 | -0.023 | 0.005 | 7.10E-06 | -0.017 | 0.036 | 0.640 |
|  |  |  | rs7342459 |  | T | 0.968 | 0.050 | 0.011 | 5.70E-06 | 0.075 | 0.082 | 0.358 |
|  |  |  | rs7492369 | rs55695162 (0.81) | C | 0.749 | -0.020 | 0.005 | 1.40E-05 | 0.018 | 0.031 | 0.563 |
|  |  |  | rs797148 |  | C | 0.917 | -0.033 | 0.007 | 4.80E-06 | -0.064 | 0.050 | 0.206 |
|  |  |  | rs10035367 | rs7711883 (0.99) | C | 0.845 | -0.023 | 0.005 | 1.80E-05 | 0.041 | 0.038 | 0.288 |
| Insomnia | Caffeine metabolic rate | p<5×10^-8^ | rs11693221 |  | T | 0.048 | 0.171 | 0.023 | 3.79E-14 | -0.096 | 0.062 | 0.118 |
|  |  |  |  |  |  |  |  |  |  |  |  |  |
| Insomnia | Caffeine metabolic rate | p<1×10^-5^ | rs11039701 | rs1994140 (1.0) | G | 0.564 | -0.045 | 0.009 | 1.60E-06 | -0.019 | 0.028 | 0.487 |
|  |  |  | rs11653468 | rs72835405 (0.99) | A | 0.233 | -0.049 | 0.011 | 9.08E-06 | 0.019 | 0.030 | 0.521 |
|  |  |  | rs12705971 | rs10250103 (0.98) | T | 0.475 | 0.046 | 0.009 | 1.48E-06 | 0.037 | 0.026 | 0.149 |
|  |  |  | rs1372256 | rs2087000 (0.83) | T | 0.203 | 0.049 | 0.012 | 2.49E-05 | 0.004 | 0.034 | 0.904 |
|  |  |  | rs1530399 | rs7332332 (0.85) | T | 0.470 | -0.042 | 0.009 | 8.15E-06 | -0.061 | 0.028 | 0.026 |
|  |  |  | rs1547668 |  | G | 0.796 | -0.054 | 0.012 | 3.61E-06 | 0.034 | 0.032 | 0.289 |
|  |  |  | rs16966956 | rs4772694 (1.0) | G | 0.281 | -0.047 | 0.010 | 8.66E-06 | 0.026 | 0.031 | 0.397 |
|  |  |  | rs17400178 |  | T | 0.071 | 0.084 | 0.019 | 6.63E-06 | -0.021 | 0.051 | 0.675 |
|  |  |  | rs2219778 | rs149326099 (0.88) | T | 0.039 | -0.105 | 0.024 | 1.54E-05 | 0.011 | 0.068 | 0.875 |
|  |  |  | rs2673608 | rs2673609 (0.96) | C | 0.346 | 0.043 | 0.010 | 1.37E-05 | -0.029 | 0.029 | 0.305 |
|  |  |  | rs3913801 | rs6437732 (0.86) | A | 0.484 | -0.036 | 0.009 | 1.61E-04 | 0.042 | 0.027 | 0.121 |
|  |  |  | rs5752673 | rs738475 (0.83) | T | 0.242 | 0.049 | 0.011 | 7.78E-06 | -0.024 | 0.032 | 0.454 |
|  |  |  | rs641783 | rs865199 (0.96) | A | 0.065 | -0.086 | 0.019 | 7.36E-06 | 0.022 | 0.056 | 0.694 |
|  |  |  | rs9383763 | rs9383762 (0.95) | C | 0.478 | 0.037 | 0.009 | 7.97E-05 | 0.000 | 0.027 | 0.998 |

**Table S2.** Cochran's Heterogeneity statistic for Inverse Variance Weighted (IVW) bi-directional, two sample Mendelian randomization analyses between *caffeine intake* and sleep behaviours

| Exposure | Outcome | Threshold  genetic instrument | *n* SNPs | Cochran’s heterogeneity statistic | |
| --- | --- | --- | --- | --- | --- |
|  |  |  |  | Q | P |
| Caffeine intake | Sleep duration | *p*<5×10^-8^ | 4 | 1.935 | 0.586 |
| Caffeine intake | Sleep duration | *p*<1×10^-5^ | 27 | 31.236 | 0.220 |
| Caffeine intake | Chronotype | *p*<5×10^-8^ | 4 | 4.638 | 0.200 |
| Caffeine intake | Chronotype | *p*<1×10^-5^ | 27 | 47.083 | 0.007 |
| Caffeine intake | Insomnia | *p*<5×10^-8^ | 4 | 0.804 | 0.848 |
| Caffeine intake | Insomnia | *p*<1×10^-5^ | 27 | 19.315 | 0.823 |
| Sleep duration | Caffeine intake | *p*<5×10^-8^ | 3 | 0.763 | 0.683 |
| Sleep duration | Caffeine intake | *p*<1×10^-5^ | 23 | 29.522 | 0.131 |
| Chronotype | Caffeine intake | *p*<5×10^-8^ | 8 | 4.494 | 0.721 |
| Chronotype | Caffeine intake | *p*<1×10^-5^ | 55 | 54.551 | 0.453 |
| Insomnia | Caffeine intake | *p*<5×10^-8^ | 1 | *n.a.* | *n.a.* |
| Insomnia | Caffeine intake | *p*<1×10^-5^ | 16 | 12.553 | 0.637 |

n.a.: not available because the genetic instrument consists of only 1 or 2 SNPs

**Table S3.** MR-Egger intercept, indicating pleiotropy, for bi-directional, two sample Mendelian randomization analyses between *caffeine intake* and sleep behaviours.

| Exposure | Outcome | Threshold  genetic instrument | *n* SNPs | MR-Egger intercept | | |
| --- | --- | --- | --- | --- | --- | --- |
|  |  |  |  | beta | SE | P |
| Caffeine intake | Sleep duration | *p*<5×10^-8^ | 4 | *n.a.* | *n.a.* | *n.a.* |
| Caffeine intake | Sleep duration | *p*<1×10^-5^ | 27 | 0.001 | 0.002 | 0.760 |
| Caffeine intake | Chronotype | *p*<5×10^-8^ | 4 | *n.a.* | *n.a.* | *n.a.* |
| Caffeine intake | Chronotype | *p*<1×10^-5^ | 27 | -0.004 | 0.002 | 0.086 |
| Caffeine intake | Insomnia | *p*<5×10^-8^ | 4 | *n.a.* | *n.a.* | *n.a.* |
| Caffeine intake | Insomnia | *p*<1×10^-5^ | 27 | -0.002 | 0.004 | 0.641 |
| Sleep duration | Caffeine intake | *p*<5×10^-8^ | 3 | *n.a.* | *n.a.* | *n.a.* |
| Sleep duration | Caffeine intake | *p*<1×10^-5^ | 23 | -0.014 | 0.009 | 0.139 |
| Chronotype | Caffeine intake | *p*<5×10^-8^ | 8 | *n.a.* | *n.a.* | *n.a.* |
| Chronotype | Caffeine intake | *p*<1×10^-5^ | 55 | 0.011 | 0.005 | 0.042 |
| Insomnia | Caffeine intake | *p*<5×10^-8^ | 1 | *n.a.* | *n.a.* | *n.a.* |
| Insomnia | Caffeine intake | *p*<1×10^-5^ | 16 | 0.003 | 0.010 | 0.770 |

n.a.: not available because the genetic instrument consists of <10 SNPs

**Table S4.** Cochran's Heterogeneity statistic for Inverse Variance Weighted (IVW) bi-directional, two sample Mendelian randomization analyses between *plasma caffeine* and sleep behaviours

| Exposure | Outcome | Threshold  genetic instrument | *n* SNPs | Cochran’s heterogeneity statistic | |
| --- | --- | --- | --- | --- | --- |
|  |  |  |  | Q | P |
| Plasma caffeine | Sleep duration | *p*<5×10^-8^ | 1 | *n.a.* | *n.a.* |
| Plasma caffeine | Sleep duration | *p*<1×10^-5^ | 11 | 8.460 | 0.584 |
| Plasma caffeine | Chronotype | *p*<5×10^-8^ | 1 | *n.a.* | *n.a.* |
| Plasma caffeine | Chronotype | *p*<1×10^-5^ | 11 | 12.001 | 0.285 |
| Plasma caffeine | Insomnia | *p*<5×10^-8^ | 1 | *n.a.* | *n.a.* |
| Plasma caffeine | Insomnia | *p*<1×10^-5^ | 11 | 11.895 | 0.292 |
| Sleep duration | Plasma caffeine | *p*<5×10^-8^ | 2 | *n.a.* | *n.a.* |
| Sleep duration | Plasma caffeine | *p*<1×10^-5^ | 16 | 8.580 | 0.898 |
| Chronotype | Plasma caffeine | *p*<5×10^-8^ | 4 | 0.880 | 0.830 |
| Chronotype | Plasma caffeine | *p*<1×10^-5^ | 42 | 33.764 | 0.781 |
| Insomnia | Plasma caffeine | *p*<5×10^-8^ | 1 | *n.a.* | *n.a.* |
| Insomnia | Plasma caffeine | *p*<1×10^-5^ | 14 | 9.726 | 0.716 |

n.a.: not available because the genetic instrument consists of only 1 or 2 SNPs

**Table S5.** MR-Egger intercept, indicating pleiotropy, for bi-directional, two sample Mendelian randomization analyses between *plasma caffeine* and sleep behaviours

| Exposure | Outcome | Threshold  genetic instrument | *n* SNPs | MR-Egger intercept | | |
| --- | --- | --- | --- | --- | --- | --- |
|  |  |  |  | beta | SE | P |
| Plasma caffeine | Sleep duration | *p*<5×10^-8^ | 1 | *n.a.* | *n.a.* | *n.a.* |
| Plasma caffeine | Sleep duration | *p*<1×10^-5^ | 11 | 0.004 | 0.005 | 0.413 |
| Plasma caffeine | Chronotype | *p*<5×10^-8^ | 1 | *n.a.* | *n.a.* | *n.a.* |
| Plasma caffeine | Chronotype | *p*<1×10^-5^ | 11 | 0.002 | 0.006 | 0.758 |
| Plasma caffeine | Insomnia | *p*<5×10^-8^ | 1 | *n.a.* | *n.a.* | *n.a.* |
| Plasma caffeine | Insomnia | *p*<1×10^-5^ | 11 | -0.001 | 0.013 | 0.625 |
| Sleep duration | Plasma caffeine | *p*<5×10^-8^ | 2 | *n.a.* | *n.a.* | *n.a.* |
| Sleep duration | Plasma caffeine | *p*<1×10^-5^ | 16 | -0.009 | 0.024 | 0.725 |
| Chronotype | Plasma caffeine | *p*<5×10^-8^ | 4 | *n.a.* | *n.a.* | *n.a.* |
| Chronotype | Plasma caffeine | *p*<1×10^-5^ | 42 | -0.008 | 0.016 | 0.606 |
| Insomnia | Plasma caffeine | *p*<5×10^-8^ | 1 | *n.a.* | *n.a.* | *n.a.* |
| Insomnia | Plasma caffeine | *p*<1×10^-5^ | 14 | -0.013 | 0.026 | 0.624 |

n.a.: not available because the genetic instrument consists of <10 SNPs

**Table S6.** Cochran's Heterogeneity statistic for Inverse Variance Weighted (IVW) bi-directional, two sample Mendelian randomization analyses between *caffeine metabolic rate* and sleep behaviours

| Exposure | Outcome | Threshold  genetic instrument | *n* SNPs | Cochran’s heterogeneity statistic | |
| --- | --- | --- | --- | --- | --- |
|  |  |  |  | Q | P |
| Caffeine metabolic rate | Sleep duration | *p*<5×10^-8^ | 1 | *n.a.* | *n.a.* |
| Caffeine metabolic rate | Sleep duration | *p*<1×10^-5^ | 8 | 3.907 | 0.790 |
| Caffeine metabolic rate | Chronotype | *p*<5×10^-8^ | 1 | *n.a.* | *n.a.* |
| Caffeine metabolic rate | Chronotype | *p*<1×10^-5^ | 8 | 8.873 | 0.262 |
| Caffeine metabolic rate | Insomnia | *p*<5×10^-8^ | 2 | *n.a.* | *n.a.* |
| Caffeine metabolic rate | Insomnia | *p*<1×10^-5^ | 9 | 7.510 | 0.483 |
| Sleep duration | Caffeine metabolic rate | *p*<5×10^-8^ | 2 | *n.a.* | *n.a.* |
| Sleep duration | Caffeine metabolic rate | *p*<1×10^-5^ | 16 | 14.256 | 0.506 |
| Chronotype | Caffeine metabolic rate | *p*<5×10^-8^ | 4 | 1.118 | 0.773 |
| Chronotype | Caffeine metabolic rate | *p*<1×10^-5^ | 42 | 43.328 | 0.372 |
| Insomnia | Caffeine metabolic rate | *p*<5×10^-8^ | 1 | *n.a.* | *n.a.* |
| Insomnia | Caffeine metabolic rate | *p*<1×10^-5^ | 14 | 12.800 | 0.463 |

n.a.: not available because the genetic instrument consists of only 1 or 2 SNPs

**Table S7.** MR-Egger intercept, indicating pleiotropy, for bi-directional, two sample Mendelian randomization analyses between *caffeine metabolic rate* and sleep behaviours

| Exposure | Outcome | Threshold  genetic instrument | *n* SNPs | MR-Egger intercept | | |
| --- | --- | --- | --- | --- | --- | --- |
|  |  |  |  | beta | SE | P |
| Caffeine metabolic rate | Sleep duration | *p*<5×10^-8^ | 1 | *n.a.* | *n.a.* | *n.a.* |
| Caffeine metabolic rate | Sleep duration | *p*<1×10^-5^ | 8 | *n.a.* | *n.a.* | *n.a.* |
| Caffeine metabolic rate | Chronotype | *p*<5×10^-8^ | 1 | *n.a.* | *n.a.* | *n.a.* |
| Caffeine metabolic rate | Chronotype | *p*<1×10^-5^ | 8 | *n.a.* | *n.a.* | *n.a.* |
| Caffeine metabolic rate | Insomnia | *p*<5×10^-8^ | 2 | *n.a.* | *n.a.* | *n.a.* |
| Caffeine metabolic rate | Insomnia | *p*<1×10^-5^ | 9 | *n.a.* | *n.a.* | *n.a.* |
| Sleep duration | Caffeine metabolic rate | *p*<5×10^-8^ | 2 | *n.a.* | *n.a.* | *n.a.* |
| Sleep duration | Caffeine metabolic rate | *p*<1×10^-5^ | 16 | 0.009 | 0.032 | 0.781 |
| Chronotype | Caffeine metabolic rate | *p*<5×10^-8^ | 4 | *n.a.* | *n.a.* | *n.a.* |
| Chronotype | Caffeine metabolic rate | *p*<1×10^-5^ | 42 | -0.013 | 0.022 | 0.547 |
| Insomnia | Caffeine metabolic rate | *p*<5×10^-8^ | 1 | *n.a.* | *n.a.* | *n.a.* |
| Insomnia | Caffeine metabolic rate | *p*<1×10^-5^ | 14 | 0.012 | 0.036 | 0.739 |

n.a.: not available because the genetic instrument consists of <10 SNPs
